# Supplementary material for: Towards soil-transmitted helminths transmission interruption: The impact of diagnostic tools on infection prediction in a low intensity setting in Southern Mozambique
Source: PLoS Negl Trop Dis. 2021 Oct 25;15(10):e0009803. doi: 10.1371/journal.pntd.0009803 (PMC8568186; doi:10.1371/journal.pntd.0009803)
Supplement: S1 Table — (DOCX) [file pntd.0009803.s001.docx]

S1 Table. Oligonucleotide primers and detection probes for multiplex real-time PCR for the simultaneous detection of soil-transmitted helminths.

| **Target specie** | **Oligo name** | **Oligonucleotide sequences** | **Reference** |
| --- | --- | --- | --- |
| *Trichuris trichiura* | Tt_283F | 5’- TTGAAACGACTTGCTCATCAACTT -3’ | (Liu *et al.*, 2013) [1] |
|  | Tt_358R | 5’- CTGATTCTCCGTTAACCGTTGTC -3’ |  |
|  | Tt_308T_YY | Yakima Yellow-5’- CGATGGTACGCTACGTGCTTACCATGG -3’-BHQ1 |  |
| *Ancylostoma* sp. | Ad_125F | 5’- GAATGACAGCAAACTGCTTGTTG -3’ | (Hamid *et al.*, 2011; Verweij *et al.*, 2009; Wiria *et al.*, 2010) [2-4] |
|  | Ad_195R | 5’- ATACTAGCCACTGCCGAAACGT -3’ |  |
|  | Ad_155_XS_TR | Texas red-5’- ATCGTTTACCGACTTTAG -3’BHQ2 |  |
| *Necator americanus* | Na_58F | 5’- CTGGTTTGTCGAACGGTACTTGC -3’ |  |
|  | Na_158R | 5’- ATAACAGCGTGCACATGTTGC -3’ |  |
|  | Na_81T_XS_FAM | FAM-5’- CTGTACTACGCATTGTATAC -3’-BHQ1 |  |
| *Ascaris lumbricoides* | Alum_96F | 5’- GTAATAGCAGTCGGCGGTTTCTT -3’ |  |
|  | Alum_183R | 5’- GCCCAACATGCCACCTATTC -3’ |  |
|  | Alum_124T_YY | Yakima Yellow-5’- TTGGCGGACAATTGCATGCGAT -3’-BHQ1 |  |
|  | PhHV_337as | 5’- GCGGTTCCAAACGTACCAA -3’ |  |
|  | PhHV_305tq_Cy5 | Cy5-5’- TTTTTATGTGTCCGCCACCATCTGGATC -3’-BHQ2 |  |

**References**

1. Liu, J., Gratz, J., Amour, C., Kibiki, G., Becker, S., Janaki, L., Verweij, J. J., Taniuchi, M., Sobuz, S. U., Haque, R., Haverstick, D. M. and Houpt, E. R. (2013). A laboratory-developed TaqMan Array Card for simultaneous detection of 19 enteropathogens. Journal of Clinical Microbiology 51, 472–480.

2. Hamid, F., Wiria, A. E., Wammes, L. J., Kaisar, M. M., Lell, B., Ariawan, I., Uh, H. W., Wibowo, H., Djuardi, Y., Wahyuni, S., Schot, R., Verweij, J. J., van Ree, R., May, L., Sartono, E., Yazdanbakhsh, M. and Supali, T. (2011). A longitudinal study of allergy and intestinal helminth infections in semi urban and rural areas

of Flores, Indonesia (ImmunoSPIN Study). BMC Infectious Diseases 11, 83.

3. Verweij, J. J., Canales, M., Polman, K., Ziem, J., Brienen, E. A., Polderman, A. M. and van Lieshout, L. (2009). Molecular diagnosis of Strongyloides stercoralis in faecal samples using real-time PCR. Transactions of the Royal Society of Tropical Medicine and Hygiene 103, 342–346.

4. Wiria, A. E., Prasetyani, M. A., Hamid, F., Wammes, L. J., Lell, B., Ariawan, I., Uh, H. W., Wibowo, H., Djuardi, Y., Wahyuni, S., Sutanto, I., May, L., Luty, A. J., Verweij, J. J., Sartono, E., Yazdanbakhsh, M. and Supali, T. (2010). Does treatment of intestinal helminth infections influence malaria? Background and methodology of a longitudinal study of clinical, parasitological and immunological parameters in Nangapanda, Flores, Indonesia (ImmunoSPIN Study). BMC

Infectious Diseases 10, 77.
