# Supplementary material for: Towards soil-transmitted helminths transmission interruption: The impact of diagnostic tools on infection prediction in a low intensity setting in Southern Mozambique
Source: PLoS Negl Trop Dis. 2021 Oct 25;15(10):e0009803. doi: 10.1371/journal.pntd.0009803 (PMC8568186; doi:10.1371/journal.pntd.0009803)
Supplement: S2 Table — (DOCX) [file pntd.0009803.s002.docx]

S2 Table. Potential predictors for human soil-transmitted helminths infection considered for the negative binomial model.

| Variable | Included in the model | Source |
| --- | --- | --- |
| **Land surface temperature** |  | NASA LP DAAC: MOD11A2 Land Surface Temperature and Emissivity (<https://lpdaac.usgs.gov>) [1] |
| Mean of land surface temperature | X |  |
| Minimum of land surface temperature |  |  |
| Maximum of land surface temperature |  |  |
| **Vegetation indices** |  |  |
| Mean of normalized difference vegetation index (NDVI) | X | NASA LP DAAC: MOD13Q1 Vegetation Indices (<https://lpdaac.usgs.gov>) [2] |
| Mean of enhanced vegetation index (EVI) |  |  |
| Mean of middle-infrared (MIR) |  |  |
| **Top soil pH** | X | Heng et al (2017) [3] |
| **Top soil composition** |  |  |
| Clay fraction |  | Heng et al (2017) [3] |
| Silt fraction |  |  |
| Sand fraction | X |  |
| **Population density** |  |  |
| Number of households located in a radius of < 1km around each household | X | DSS^†^ |
| Number of residents per household |  | DSS^†^ |
| **Household socioeconomical index** |  |  |
| Socioeconomical score |  | DSS^†^ |
| Head of the household education |  | DSS^†^ |
| Head of the household occupation |  | DSS^†^ |
| **Household water condition** |  |  |
| Type of main water source used (observational) |  | DSS^†^ |
| Water accessibility* | X | DSS^†^ |
| **Household sanitation conditions** |  |  |
| Owing a latrine (observational) | X | DSS^†^ |
| Type of main sanitation facility used (observational) |  | DSS^†^ |
| Latrine faeces treatment |  | DSS^†^ |
| Latrine cleanliness (observational) |  | DSS^†^ |
| **Hygiene** |  |  |
| Having a water and soap disposal next to the latrine (observational) |  | DSS |
| Participant wearing shoes the day of the visit (observational) |  | Study questionnaire |
| Presence of faeces in the yard (observational) |  | Study questionnaire |
| **Inhabitants’ characteristics** |  |  |
| Age | X | Study questionnaire |
| Gender | X | Study questionnaire |

* categorize variable composed by “having water inside the household” or “outside the household” and “being this water free” or “having to pay for water.”

^†^ Collected data during demographic surveillance system (DSS) annual census from Centro de Investigação em Saúde da Manhiça, Mozambique.

***References:***

1.NASA LP DAAC: MOD11A2 Land Surface Temperature and Emissivity 8-Day L3 Global 1km [Internet]. NASA EOSDIS Land Processes DAAC, USGS Earth Resources Observation and Science (EROS) Center, Sioux Falls, South Dakota (https://lpdaac.usgs.gov).

2. NASA LP DAAC: MOD13Q1 Vegetation Indices 16-Day L3 Global 250m [Internet]. NASA EOSDIS Land Processes DAAC, USGS Earth Resources Observation and Science (EROS) Center, Sioux Falls, South Dakota (https://lpdaac.usgs.gov).

3. Hengl T, Mendes De Jesus, J., Heuvelink, G. B. M., Ruiperez Gonzalez, M., Kilibarda, M., Blagotić, A., Shangguan, W., Wright, M. N., Geng, X., Bauer-Marschallinger, B., Guevara, M. A., Vargas, R., Macmillan, R. A., Batjes, N. H., Leenaars, J. G. B., Ribeiro, E., Wheeler, I., Mantel, S. & Kempen, B. . SoilGrids250m: Global gridded soil information based on machine learning. PloS one. 2017;12(e0169748).
