# Supplementary material for: Towards soil-transmitted helminths transmission interruption: The impact of diagnostic tools on infection prediction in a low intensity setting in Southern Mozambique
Source: PLoS Negl Trop Dis. 2021 Oct 25;15(10):e0009803. doi: 10.1371/journal.pntd.0009803 (PMC8568186; doi:10.1371/journal.pntd.0009803)
Supplement: S3 Table — (DOCX) [file pntd.0009803.s003.docx]

S3 Table. Sensitivity and 95% confidence intervals [95% CI] of Telemann in one or two stool samples, single and duplicate Kato-Katz in one stool sample, single and duplicate Kato-Katz in two stool samples, multiplex quantitative PCR compared to the composite reference standard (CRS) per *A. lumbricoides, T. trichiura* and hookworm.

|  | 1. ***lumbricoides*** | ***T. trichiura*** | **Hookworm** |
| --- | --- | --- | --- |
|  | **Sensitivity (%)**  **(95% CI)** | **Sensitivity (%)**  **(95% CI)** | **Sensitivity (%)**  **(95% CI)** |
| CRS | - | - | - |
| Telemann x1 ^β^ | 42.2 (29.9-55.2) | 35.8 (26.8-45.5) | 43.2 (36.8-49.8) |
| Telemann x2 ^†^ | 57.8 (44.8-70.1) | 46.8 (37.2-56.6) | 55.10 (48.5-61.5) |
| Single Kato-Katz x1 ^β^ | 34.4 (22.9-47.3) | 29.4 (21.0-38.8) | 29.2 (23.5-35.5) |
| Duplicate Kato-Katz x1 ^β^ | 39.1 (27.1-52.1) | 35.8 (26.8-45.5) | 34.3 (28.3-40.8) |
| Single Kato-Katz x2 ^†^ | 48.4 (35.8-61.3) | 41.3 (31.9-51.1) | 42.4 (36.0-49.0) |
| Duplicate Kato-Katz x2 ^†^ | 53.1 (40.2-65.7) | 47.7 (38.1-57.5) | 48.3 (41.8-54.9) |
| qPCR ^β^ | 92.2 (82.7-97.4) | 94.5 (88.4-98.0) | 94.9 (91.3-97.3) |

^β^ from one stool sample per participant

^†^ from two consecutive stool samples per participant
