# Supplementary material for: Towards soil-transmitted helminths transmission interruption: The impact of diagnostic tools on infection prediction in a low intensity setting in Southern Mozambique
Source: PLoS Negl Trop Dis. 2021 Oct 25;15(10):e0009803. doi: 10.1371/journal.pntd.0009803 (PMC8568186; doi:10.1371/journal.pntd.0009803)
Supplement: S4 Table — (DOCX) [file pntd.0009803.s004.docx]

S4 Table. Estimated prevalence of at least one STH infection and its associated standard error and confidence interval (95%) per region using Telemann from one or two stools, single Kato-Katz from one or two stool samples, duplicate Kato-Katz from one or two stools and qPCR.

| **Region** | **Telemann x1** | | **Telemann x2** | | **Single KK x1** | | **Duplicate KK x1** | | **Single KK x2** | | **Dupliacte KK x2** | | **qPCR** | |
| --- | --- | --- | --- | --- | --- | --- | --- | --- | --- | --- | --- | --- | --- | --- |
|  | **Estimated prevalence (%)** | **Standard error (%)** | **Estimated prevalence (%)** | **Standard error (%)** | **Estimated prevalence (%)** | **Standard error (%)** | **Estimated prevalence (%)** | **Standard error (%)** | **Estimated prevalence (%)** | **Standard error (%)** | **Estimated prevalence (%)** | **Standard error (%)** | **Estimated prevalence (%)** | **Standard error (%)** |
| **1** | 8.6 | 4.8 | 16.1 | 5.9 | 5.7 | 4.0 | 5.9 | 4.0 | 11.6 | 4.9 | 12.3 | 5.3 | 34.7 | 7.8 |
| **2** | 9.3 | 4.5 | 16.5 | 5.3 | 6.1 | 3.7 | 6.5 | 3.8 | 12.3 | 4.5 | 13.3 | 5.0 | 34.7 | 6.9 |
| **3** | 9.7 | 4.2 | 17.4 | 5.0 | 6.1 | 3.3 | 6.9 | 3.5 | 12.3 | 4.1 | 14.2 | 4.7 | 37.5 | 6.4 |
| **4** | 10.2 | 3.8 | 17.7 | 4.5 | 7.5 | 3.3 | 7.9 | 3.3 | 12.5 | 3.6 | 14.1 | 4.1 | 38.2 | 5.6 |
| **5** | 11.4 | 3.9 | 19.1 | 4.3 | 8.0 | 3.3 | 8.8 | 3.4 | 14.6 | 3.9 | 15.7 | 4.1 | 41.0 | 5.3 |
| **6** | 7.6 | 3.5 | 14.4 | 4.4 | 5.5 | 3.1 | 5.6 | 3.0 | 10.1 | 3.6 | 11.0 | 3.9 | 32.6 | 6.1 |
| **7** | 9.0 | 3.0 | 15.7 | 3.5 | 6.4 | 2.5 | 7.0 | 2.5 | 11.6 | 3.1 | 12.6 | 3.3 | 35.0 | 4.7 |
| **8** | 9.5 | 5.1 | 16.8 | 5.9 | 6.1 | 4.1 | 6.6 | 4.3 | 12.6 | 5.1 | 13.6 | 5.6 | 33.9 | 7.5 |
| **9** | 8.2 | 5.1 | 15.6 | 6.3 | 6.1 | 4.6 | 5.8 | 4.3 | 10.8 | 4.9 | 11.5 | 5.5 | 32.7 | 8.2 |
| **10** | 7.9 | 5.2 | 15.4 | 6.6 | 5.7 | 4.6 | 5.5 | 4.4 | 10.7 | 5.2 | 11.5 | 5.8 | 33.1 | 8.7 |
| **11** | 7.8 | 5.3 | 15.5 | 6.8 | 5.6 | 4.7 | 5.3 | 4.4 | 11.1 | 5.5 | 11.6 | 5.9 | 33.3 | 9.0 |
| **12** | 7.4 | 5.3 | 14.9 | 6.8 | 5.3 | 4.6 | 5.0 | 4.3 | 10.8 | 5.6 | 10.9 | 5.9 | 31.9 | 9.1 |
| **13** | 7.4 | 5.5 | 14.9 | 7.0 | 5.4 | 4.9 | 5.1 | 4.5 | 11.1 | 5.9 | 11.1 | 6.2 | 32.0 | 9.4 |
| **14** | 8.4 | 5.1 | 15.4 | 6.1 | 6.6 | 4.7 | 6.2 | 4.3 | 11.3 | 4.9 | 11.6 | 5.3 | 31.2 | 7.8 |
| **15** | 7.7 | 5.3 | 14.9 | 6.6 | 5.8 | 4.8 | 5.4 | 4.4 | 10.6 | 5.3 | 11.0 | 5.7 | 31.1 | 8.7 |
| **16** | 7.3 | 5.6 | 14.6 | 7.1 | 5.4 | 5.0 | 5.0 | 4.6 | 10.3 | 5.7 | 10.4 | 6.0 | 31.2 | 9.5 |
| **17** | 6.6 | 5.3 | 13.4 | 6.9 | 4.9 | 4.8 | 4.5 | 4.4 | 10.1 | 5.9 | 9.7 | 6.0 | 28.9 | 9.5 |
| **18** | 10.0 | 5.2 | 16.7 | 5.7 | 6.1 | 3.8 | 6.9 | 4.3 | 13.6 | 5.3 | 14.2 | 5.7 | 31.5 | 7.0 |
| **19** | 8.0 | 4.0 | 13.6 | 4.7 | 5.8 | 3.6 | 5.8 | 3.5 | 11.1 | 4.3 | 11.3 | 4.5 | 27.0 | 6.3 |
| **20** | 9.3 | 4.4 | 15.4 | 5.2 | 5.7 | 3.3 | 6.5 | 3.6 | 10.2 | 4.0 | 13.0 | 4.8 | 29.6 | 6.9 |
| **21** | 7.5 | 4.9 | 14.0 | 6.0 | 5.9 | 4.6 | 5.4 | 4.2 | 10.9 | 5.2 | 10.7 | 5.4 | 28.4 | 7.9 |
| **22** | 8.8 | 5.1 | 15.5 | 6.1 | 6.8 | 4.7 | 6.4 | 4.4 | 11.6 | 5.0 | 11.8 | 5.3 | 29.8 | 7.6 |
| **23** | 7.2 | 4.7 | 13.8 | 5.9 | 4.8 | 4.1 | 5.0 | 3.9 | 10.6 | 5.3 | 11.8 | 5.8 | 30.7 | 8.3 |
| **24** | 9.0 | 4.9 | 15.7 | 5.8 | 7.4 | 4.6 | 6.8 | 4.2 | 11.1 | 4.5 | 11.9 | 5.0 | 31.0 | 7.4 |
| **25** | 6.4 | 5.7 | 12.9 | 7.2 | 4.5 | 5.0 | 4.1 | 4.6 | 10.2 | 6.5 | 9.4 | 6.3 | 26.9 | 10.0 |
| **26** | 7.2 | 5.8 | 14.1 | 7.2 | 5.4 | 5.2 | 4.9 | 4.7 | 10.6 | 6.2 | 10.2 | 6.3 | 28.3 | 9.6 |
| **27** | 7.4 | 5.2 | 14.1 | 6.5 | 5.3 | 4.6 | 5.0 | 4.3 | 10.6 | 5.6 | 11.0 | 5.9 | 29.5 | 8.8 |
| **28** | 7.2 | 5.6 | 14.1 | 7.1 | 5.4 | 5.2 | 4.9 | 4.7 | 11.2 | 6.3 | 10.9 | 6.4 | 29.5 | 9.6 |
| **29** | 6.4 | 5.3 | 12.8 | 6.8 | 5.2 | 5.1 | 4.5 | 4.5 | 9.8 | 5.9 | 9.0 | 5.8 | 26.8 | 9.3 |
| **30** | 5.6 | 4.7 | 11.4 | 6.1 | 3.9 | 4.1 | 3.7 | 3.9 | 8.9 | 5.5 | 8.3 | 5.4 | 24.2 | 8.9 |
| **31** | 4.6 | 3.9 | 9.8 | 5.4 | 3.2 | 3.6 | 3.1 | 3.2 | 6.9 | 4.7 | 7.9 | 5.1 | 24.3 | 8.7 |
| **32** | 5.5 | 4.5 | 10.9 | 5.8 | 3.3 | 3.6 | 3.5 | 3.6 | 7.6 | 4.9 | 8.6 | 5.4 | 24.0 | 8.6 |
| **33** | 5.5 | 5.0 | 11.0 | 6.4 | 4.1 | 4.6 | 3.7 | 4.1 | 8.1 | 5.4 | 7.5 | 5.3 | 22.6 | 9.0 |
| **34** | 6.0 | 3.8 | 11.5 | 4.9 | 4.4 | 3.4 | 4.3 | 3.2 | 8.7 | 4.2 | 8.3 | 4.2 | 25.2 | 7.1 |
| **35** | 5.6 | 4.3 | 11.0 | 5.4 | 4.1 | 3.8 | 3.9 | 3.6 | 8.3 | 4.6 | 8.0 | 4.7 | 22.8 | 7.7 |
| **36** | 5.8 | 4.1 | 11.5 | 5.3 | 4.0 | 3.5 | 4.0 | 3.5 | 8.4 | 4.5 | 8.5 | 4.6 | 24.8 | 7.6 |
| **37** | 5.6 | 4.8 | 11.1 | 6.1 | 3.9 | 4.1 | 3.8 | 4.0 | 8.2 | 5.2 | 8.0 | 5.2 | 23.0 | 8.6 |
| **38** | 5.6 | 4.6 | 11.2 | 6.0 | 4.0 | 4.1 | 3.8 | 3.9 | 8.1 | 5.0 | 8.0 | 5.1 | 23.8 | 8.6 |
| **39** | 4.4 | 4.0 | 9.7 | 5.8 | 3.2 | 3.9 | 3.1 | 3.3 | 7.5 | 5.3 | 8.4 | 5.6 | 25.2 | 9.3 |
| **40** | 5.8 | 4.9 | 11.4 | 6.2 | 4.5 | 4.6 | 4.0 | 4.1 | 9.1 | 5.6 | 8.2 | 5.4 | 23.3 | 8.7 |
| **41** | 6.0 | 4.5 | 11.8 | 5.7 | 4.5 | 4.1 | 4.2 | 3.8 | 8.8 | 4.8 | 8.4 | 4.9 | 24.8 | 8.1 |
| **42** | 6.0 | 3.9 | 11.7 | 5.0 | 4.3 | 3.4 | 4.3 | 3.3 | 8.0 | 4.0 | 9.0 | 4.4 | 25.8 | 7.2 |
| **43** | 6.8 | 3.8 | 12.4 | 4.7 | 4.8 | 3.2 | 5.1 | 3.3 | 7.9 | 3.5 | 9.9 | 4.2 | 27.1 | 6.6 |
| **44** | 7.3 | 3.6 | 12.3 | 4.3 | 3.7 | 2.6 | 4.9 | 3.0 | 8.3 | 3.5 | 10.8 | 4.3 | 25.5 | 6.1 |
| **45** | 7.2 | 3.7 | 13.0 | 4.5 | 4.9 | 3.1 | 5.2 | 3.2 | 9.2 | 3.7 | 10.5 | 4.2 | 28.5 | 6.3 |
| **46** | 6.4 | 4.1 | 12.2 | 5.1 | 4.3 | 3.4 | 4.4 | 3.4 | 8.9 | 4.3 | 9.1 | 4.5 | 26.5 | 7.3 |
| **47** | 7.7 | 3.3 | 14.2 | 4.1 | 5.1 | 2.7 | 5.6 | 2.8 | 10.2 | 3.5 | 11.1 | 3.8 | 32.6 | 5.9 |
| **48** | 6.9 | 4.4 | 13.0 | 5.5 | 5.3 | 4.0 | 5.0 | 3.7 | 8.6 | 4.1 | 9.3 | 4.5 | 27.1 | 7.4 |
| **49** | 10.8 | 2.4 | 16.9 | 2.9 | 4.7 | 1.5 | 7.5 | 2.1 | 12.2 | 2.6 | 15.5 | 2.9 | 37.5 | 3.9 |
| **50** | 9.7 | 2.4 | 15.8 | 2.9 | 6.6 | 2.0 | 7.9 | 2.0 | 10.4 | 2.3 | 12.9 | 2.6 | 36.3 | 3.9 |
| **51** | 14.8 | 2.4 | 21.0 | 2.7 | 8.4 | 1.8 | 11.8 | 2.3 | 15.3 | 2.4 | 19.4 | 2.6 | 42.9 | 3.3 |
| **52** | 8.9 | 3.7 | 16.1 | 4.4 | 5.7 | 3.0 | 6.6 | 3.2 | 11.7 | 3.8 | 13.0 | 4.2 | 36.0 | 5.9 |
| **53** | 10.7 | 3.4 | 18.3 | 3.9 | 6.4 | 2.5 | 8.0 | 2.9 | 13.1 | 3.3 | 15.9 | 3.8 | 40.7 | 4.9 |
| **54** | 12.8 | 3.1 | 20.1 | 3.5 | 6.7 | 2.1 | 9.7 | 2.7 | 14.4 | 3.2 | 18.7 | 3.6 | 43.9 | 4.5 |
| **55** | 10.7 | 3.7 | 18.0 | 4.3 | 9.3 | 3.4 | 9.3 | 3.2 | 12.1 | 3.5 | 14.2 | 4.0 | 40.0 | 5.4 |
| **56** | 13.1 | 4.5 | 19.6 | 4.8 | 12.2 | 4.3 | 11.8 | 4.0 | 15.3 | 4.3 | 15.8 | 4.6 | 38.0 | 5.6 |
| **57** | 5.9 | 3.8 | 11.4 | 5.0 | 4.7 | 3.7 | 4.6 | 3.3 | 9.5 | 4.8 | 10.3 | 5.1 | 28.6 | 7.6 |
| **58** | 10.9 | 4.9 | 18.0 | 5.5 | 9.6 | 4.8 | 8.9 | 4.3 | 14.3 | 4.8 | 13.7 | 5.0 | 36.0 | 6.6 |
| **59** | 9.2 | 4.6 | 16.8 | 5.6 | 7.0 | 4.1 | 6.9 | 3.8 | 11.3 | 4.3 | 12.8 | 4.9 | 35.9 | 7.2 |
| **60** | 7.5 | 6.1 | 14.9 | 7.6 | 5.4 | 5.3 | 5.0 | 4.9 | 11.5 | 6.7 | 11.0 | 6.7 | 30.7 | 10.1 |
| **61** | 8.5 | 4.9 | 16.0 | 6.0 | 6.2 | 4.2 | 5.9 | 4.0 | 11.4 | 4.9 | 11.9 | 5.2 | 33.6 | 7.8 |
| **62** | 11.2 | 3.9 | 19.6 | 4.6 | 8.0 | 3.4 | 8.6 | 3.4 | 13.7 | 3.8 | 15.6 | 4.3 | 43.2 | 5.6 |
| **63** | 7.2 | 6.0 | 14.0 | 7.4 | 5.2 | 5.2 | 4.7 | 4.8 | 12.3 | 7.5 | 10.4 | 6.8 | 28.3 | 10.2 |
| **64** | 7.1 | 4.6 | 13.7 | 5.7 | 5.4 | 4.1 | 4.9 | 3.7 | 10.8 | 5.1 | 10.0 | 5.0 | 29.4 | 8.0 |
| **65** | 8.6 | 3.7 | 15.8 | 4.5 | 6.5 | 3.3 | 6.4 | 3.2 | 11.6 | 3.9 | 11.8 | 4.1 | 35.5 | 6.1 |
| **66** | 8.9 | 3.1 | 15.7 | 3.7 | 6.5 | 2.7 | 6.9 | 2.7 | 11.1 | 3.1 | 12.1 | 3.4 | 35.9 | 5.0 |
| **67** | 8.4 | 2.8 | 15.0 | 3.5 | 6.5 | 2.5 | 6.8 | 2.4 | 9.7 | 2.7 | 11.4 | 3.1 | 35.1 | 4.7 |
| **68** | 7.6 | 3.6 | 14.3 | 4.5 | 6.0 | 3.3 | 5.7 | 3.0 | 10.6 | 3.8 | 10.4 | 3.9 | 32.0 | 6.4 |
| **69** | 8.6 | 2.4 | 14.5 | 3.0 | 6.5 | 2.2 | 6.9 | 2.1 | 10.1 | 2.4 | 11.2 | 2.6 | 32.3 | 4.0 |
| **70** | 8.1 | 3.0 | 14.5 | 3.6 | 5.8 | 2.6 | 6.1 | 2.5 | 10.5 | 3.0 | 11.3 | 3.2 | 32.2 | 5.0 |
| **71** | 8.9 | 2.4 | 14.9 | 3.0 | 6.8 | 2.1 | 7.4 | 2.1 | 9.7 | 2.3 | 11.6 | 2.6 | 34.5 | 4.0 |
| **72** | 9.8 | 2.4 | 15.6 | 3.0 | 7.6 | 2.3 | 8.2 | 2.1 | 11.0 | 2.5 | 12.3 | 2.6 | 35.4 | 4.0 |
| **73** | 10.9 | 3.0 | 16.7 | 3.6 | 9.1 | 2.9 | 9.7 | 2.8 | 12.1 | 3.1 | 13.2 | 3.1 | 37.7 | 4.7 |
| **74** | 10.7 | 2.7 | 16.4 | 3.2 | 8.7 | 2.6 | 9.4 | 2.5 | 12.2 | 2.8 | 13.2 | 2.8 | 36.3 | 4.2 |
| **75** | 12.4 | 1.8 | 18.3 | 2.2 | 7.4 | 1.5 | 9.8 | 1.7 | 13.4 | 1.9 | 16.2 | 2.0 | 38.5 | 2.8 |
| **76** | 11.4 | 2.0 | 17.2 | 2.4 | 7.2 | 1.7 | 9.4 | 1.9 | 12.6 | 2.1 | 15.2 | 2.2 | 38.1 | 3.1 |
| **77** | 10.3 | 2.5 | 16.2 | 3.0 | 8.1 | 2.4 | 8.9 | 2.3 | 11.8 | 2.6 | 13.2 | 2.7 | 36.6 | 4.0 |
| **78** | 9.0 | 2.4 | 14.5 | 3.0 | 7.5 | 2.3 | 7.7 | 2.1 | 10.5 | 2.5 | 11.4 | 2.6 | 32.9 | 4.0 |
| **79** | 12.1 | 1.8 | 17.6 | 2.1 | 7.8 | 1.6 | 9.8 | 1.6 | 13.1 | 1.8 | 15.3 | 1.9 | 37.0 | 2.7 |
| **80** | 11.8 | 2.2 | 17.7 | 2.6 | 8.2 | 2.1 | 9.9 | 2.1 | 12.8 | 2.3 | 15.0 | 2.3 | 38.6 | 3.4 |
| **81** | 10.4 | 2.0 | 16.1 | 2.4 | 7.8 | 1.9 | 8.8 | 1.8 | 11.8 | 2.1 | 13.4 | 2.1 | 36.1 | 3.2 |
| **82** | 9.8 | 2.8 | 15.5 | 3.4 | 8.5 | 2.7 | 8.7 | 2.5 | 11.3 | 3.0 | 12.0 | 3.0 | 35.7 | 4.5 |
| **83** | 11.5 | 2.1 | 17.2 | 2.5 | 8.5 | 2.0 | 9.7 | 2.0 | 12.9 | 2.2 | 14.4 | 2.2 | 36.7 | 3.3 |
| **84** | 11.0 | 1.9 | 15.0 | 2.2 | 5.9 | 1.4 | 8.3 | 1.7 | 10.6 | 1.9 | 13.7 | 2.1 | 30.3 | 2.8 |
| **85** | 12.5 | 1.7 | 17.0 | 2.1 | 7.3 | 1.4 | 9.6 | 1.6 | 11.8 | 1.7 | 14.8 | 1.9 | 32.6 | 2.6 |
| **86** | 11.7 | 1.7 | 17.1 | 2.1 | 7.5 | 1.5 | 9.4 | 1.6 | 12.3 | 1.8 | 14.9 | 1.9 | 36.3 | 2.7 |
| **87** | 7.3 | 5.7 | 13.7 | 6.9 | 5.3 | 5.0 | 5.0 | 4.8 | 10.2 | 5.8 | 10.1 | 6.0 | 26.6 | 8.9 |
| **88** | 6.3 | 6.9 | 12.5 | 8.6 | 4.4 | 6.0 | 4.1 | 5.7 | 10.3 | 8.3 | 9.3 | 7.8 | 23.9 | 11.3 |
| **89** | 8.1 | 5.4 | 14.9 | 6.5 | 5.8 | 4.8 | 5.7 | 4.6 | 11.2 | 5.6 | 11.2 | 5.8 | 30.4 | 8.5 |
| **90** | 6.9 | 5.0 | 12.7 | 6.1 | 5.2 | 4.5 | 4.9 | 4.2 | 10.0 | 5.3 | 9.4 | 5.3 | 25.7 | 8.2 |
| **91** | 7.2 | 4.5 | 13.1 | 5.5 | 5.0 | 3.8 | 5.2 | 3.7 | 9.7 | 4.7 | 9.9 | 4.8 | 26.9 | 7.5 |
| **92** | 7.5 | 4.4 | 13.7 | 5.4 | 5.4 | 3.9 | 5.5 | 3.8 | 10.6 | 4.8 | 10.4 | 4.9 | 28.9 | 7.5 |
| **93** | 8.2 | 4.1 | 14.6 | 4.8 | 5.3 | 3.3 | 5.8 | 3.5 | 10.7 | 4.1 | 11.2 | 4.5 | 30.4 | 6.6 |
| **94** | 8.5 | 3.4 | 15.0 | 4.0 | 5.6 | 2.7 | 6.3 | 2.9 | 9.9 | 3.1 | 11.6 | 3.6 | 32.9 | 5.4 |
| **95** | 9.9 | 3.1 | 16.5 | 3.5 | 6.6 | 2.5 | 7.6 | 2.7 | 11.4 | 2.9 | 13.2 | 3.3 | 36.1 | 4.7 |
| **96** | 11.3 | 2.8 | 18.1 | 3.2 | 7.0 | 2.1 | 8.6 | 2.4 | 11.8 | 2.6 | 14.5 | 2.9 | 39.4 | 4.1 |
| **97** | 13.1 | 2.7 | 20.5 | 3.2 | 8.4 | 2.2 | 10.3 | 2.4 | 12.6 | 2.5 | 16.7 | 2.9 | 44.1 | 3.9 |
| **98** | 15.3 | 3.1 | 21.8 | 3.9 | 7.5 | 2.2 | 10.8 | 2.8 | 14.1 | 3.2 | 18.8 | 3.5 | 42.3 | 4.6 |
| **99** | 6.2 | 7.6 | 12.2 | 9.4 | 4.2 | 6.7 | 4.0 | 6.4 | 10.7 | 9.6 | 9.2 | 8.8 | 23.1 | 12.5 |
| **100** | 5.6 | 7.4 | 11.2 | 9.1 | 4.0 | 6.6 | 3.6 | 6.2 | 10.4 | 9.7 | 8.3 | 8.6 | 21.3 | 12.4 |
| **101** | 5.5 | 5.9 | 11.0 | 7.4 | 3.9 | 5.1 | 3.6 | 4.9 | 9.7 | 7.6 | 8.1 | 6.9 | 21.8 | 10.5 |
| **102** | 6.6 | 3.2 | 12.1 | 4.0 | 4.3 | 2.6 | 4.8 | 2.8 | 9.1 | 3.5 | 9.4 | 3.7 | 26.8 | 6.1 |
| **103** | 9.1 | 2.0 | 14.6 | 2.5 | 6.0 | 1.7 | 7.3 | 1.7 | 9.4 | 1.9 | 11.9 | 2.2 | 33.0 | 3.4 |
| **104** | 6.8 | 2.6 | 12.5 | 3.4 | 4.9 | 2.2 | 5.4 | 2.2 | 7.5 | 2.4 | 9.2 | 2.9 | 29.5 | 4.9 |
| **105** | 6.0 | 3.7 | 11.7 | 4.8 | 4.2 | 3.2 | 4.3 | 3.2 | 8.1 | 3.9 | 8.5 | 4.2 | 26.0 | 7.1 |
| **106** | 5.5 | 5.3 | 11.0 | 6.8 | 3.8 | 4.6 | 3.6 | 4.4 | 9.0 | 6.5 | 7.9 | 6.1 | 22.6 | 9.8 |
| **107** | 5.2 | 6.5 | 10.5 | 8.2 | 3.8 | 6.0 | 3.4 | 5.5 | 9.1 | 8.3 | 7.5 | 7.4 | 20.5 | 11.3 |
| **108** | 6.3 | 7.5 | 12.2 | 9.1 | 4.1 | 6.3 | 4.0 | 6.3 | 10.6 | 9.3 | 9.4 | 8.7 | 23.0 | 12.1 |
| **109** | 5.8 | 7.2 | 11.6 | 9.0 | 4.2 | 6.5 | 3.8 | 6.1 | 10.6 | 9.5 | 8.5 | 8.3 | 22.7 | 12.4 |
| **110** | 5.8 | 5.2 | 11.2 | 6.5 | 4.0 | 4.4 | 3.8 | 4.3 | 9.7 | 6.7 | 8.1 | 6.0 | 22.9 | 9.6 |
| **111** | 7.3 | 3.6 | 13.3 | 4.5 | 4.7 | 2.8 | 5.1 | 3.0 | 10.8 | 4.3 | 10.5 | 4.3 | 29.2 | 6.7 |
| **112** | 8.2 | 2.5 | 13.9 | 3.1 | 5.6 | 2.2 | 6.2 | 2.2 | 10.1 | 2.7 | 10.8 | 2.7 | 31.2 | 4.6 |
| **113** | 7.5 | 2.7 | 13.1 | 3.4 | 5.5 | 2.4 | 5.8 | 2.3 | 9.4 | 2.9 | 9.7 | 2.9 | 30.0 | 5.1 |
| **114** | 15.2 | 2.0 | 20.2 | 2.3 | 9.0 | 1.7 | 12.3 | 2.0 | 16.0 | 2.1 | 18.7 | 2.2 | 38.2 | 2.8 |
| **115** | 6.5 | 6.8 | 12.9 | 8.5 | 4.9 | 6.3 | 4.2 | 5.6 | 11.5 | 8.7 | 9.3 | 7.8 | 25.5 | 11.7 |
| **116** | 5.9 | 6.9 | 12.0 | 8.7 | 4.2 | 6.2 | 3.8 | 5.8 | 10.0 | 8.5 | 8.6 | 7.8 | 24.1 | 12.0 |
| **117** | 5.7 | 5.8 | 11.3 | 7.4 | 4.4 | 5.4 | 3.8 | 4.9 | 9.9 | 7.5 | 8.0 | 6.6 | 23.0 | 10.5 |
| **118** | 6.1 | 4.9 | 12.0 | 6.2 | 4.6 | 4.4 | 4.2 | 4.1 | 9.8 | 5.8 | 8.7 | 5.5 | 25.0 | 8.8 |
| **119** | 6.8 | 3.5 | 12.9 | 4.4 | 5.0 | 3.1 | 4.9 | 2.9 | 9.6 | 3.8 | 9.5 | 3.9 | 28.7 | 6.4 |
| **120** | 7.9 | 2.7 | 13.8 | 3.3 | 4.6 | 2.2 | 5.5 | 2.3 | 9.9 | 2.9 | 11.1 | 3.1 | 30.3 | 4.8 |
| **121** | 14.4 | 2.5 | 19.6 | 2.9 | 7.2 | 1.8 | 11.0 | 2.4 | 15.7 | 2.7 | 18.8 | 2.8 | 38.1 | 3.5 |
| **122** | 11.1 | 1.8 | 15.8 | 2.3 | 7.1 | 1.6 | 8.7 | 1.8 | 11.2 | 2.0 | 13.7 | 2.1 | 33.7 | 2.9 |
| **123** | 10.6 | 2.1 | 14.8 | 2.6 | 5.3 | 1.5 | 7.5 | 1.8 | 9.2 | 2.0 | 12.4 | 2.3 | 30.1 | 3.2 |
| **124** | 10.5 | 2.0 | 15.0 | 2.5 | 5.2 | 1.4 | 7.5 | 1.8 | 9.2 | 1.9 | 12.7 | 2.2 | 32.5 | 3.3 |
| **125** | 14.4 | 2.5 | 18.8 | 2.9 | 7.1 | 1.8 | 10.7 | 2.3 | 12.1 | 2.3 | 16.3 | 2.7 | 35.6 | 3.5 |
| **126** | 12.3 | 2.4 | 16.7 | 3.0 | 5.9 | 1.7 | 8.7 | 2.1 | 10.8 | 2.3 | 14.0 | 2.6 | 32.8 | 3.6 |
| **127** | 11.4 | 2.0 | 15.5 | 2.5 | 6.9 | 1.7 | 8.9 | 1.9 | 10.7 | 2.0 | 13.2 | 2.2 | 31.3 | 3.0 |
| **128** | 11.8 | 2.4 | 16.1 | 3.0 | 5.6 | 1.7 | 8.3 | 2.1 | 10.2 | 2.2 | 13.3 | 2.6 | 31.7 | 3.6 |
| **129** | 14.5 | 2.6 | 19.6 | 3.2 | 7.7 | 2.0 | 10.7 | 2.4 | 13.1 | 2.6 | 16.9 | 2.9 | 37.0 | 3.6 |
| **130** | 11.6 | 2.1 | 16.5 | 2.7 | 5.4 | 1.4 | 8.2 | 1.9 | 10.0 | 2.0 | 13.7 | 2.3 | 34.0 | 3.4 |
| **131** | 17.8 | 1.9 | 23.9 | 2.1 | 11.2 | 1.6 | 14.0 | 1.8 | 18.0 | 1.9 | 21.0 | 2.0 | 41.8 | 2.4 |
| **132** | 17.7 | 2.7 | 23.3 | 2.9 | 13.3 | 2.4 | 15.0 | 2.5 | 17.0 | 2.7 | 20.4 | 2.9 | 39.0 | 3.3 |
| **133** | 16.0 | 2.1 | 22.0 | 2.4 | 9.9 | 1.8 | 12.5 | 1.9 | 16.3 | 2.1 | 19.3 | 2.3 | 39.3 | 2.8 |
| **134** | 16.7 | 2.4 | 22.5 | 2.6 | 10.9 | 2.0 | 13.1 | 2.1 | 16.2 | 2.3 | 18.9 | 2.5 | 38.9 | 3.0 |
| **135** | 15.7 | 2.1 | 21.4 | 2.3 | 10.2 | 1.7 | 12.7 | 1.9 | 15.1 | 2.0 | 18.7 | 2.2 | 37.7 | 2.8 |
| **136** | 14.5 | 1.9 | 20.4 | 2.2 | 8.4 | 1.5 | 11.0 | 1.6 | 14.1 | 1.8 | 17.6 | 2.0 | 37.6 | 2.7 |
| **137** | 10.4 | 2.0 | 15.5 | 2.6 | 5.1 | 1.3 | 7.0 | 1.7 | 9.3 | 1.9 | 12.6 | 2.2 | 30.6 | 3.4 |
| **138** | 8.3 | 2.6 | 14.1 | 3.2 | 6.2 | 2.3 | 6.8 | 2.2 | 9.7 | 2.6 | 10.8 | 2.8 | 32.7 | 4.6 |
| **139** | 11.6 | 2.9 | 18.5 | 3.3 | 8.6 | 2.5 | 9.9 | 2.6 | 12.8 | 2.8 | 15.2 | 3.1 | 41.8 | 4.1 |
| **140** | 8.4 | 2.3 | 13.5 | 2.8 | 5.9 | 2.0 | 6.8 | 2.0 | 10.0 | 2.4 | 10.9 | 2.5 | 30.0 | 4.3 |
| **141** | 11.4 | 2.6 | 17.9 | 3.0 | 8.2 | 2.2 | 9.6 | 2.3 | 12.2 | 2.5 | 14.8 | 2.7 | 39.6 | 3.8 |
| **142** | 8.3 | 2.4 | 13.7 | 3.0 | 6.0 | 2.0 | 7.0 | 2.1 | 8.8 | 2.3 | 10.7 | 2.6 | 32.0 | 4.4 |
| **143** | 10.7 | 3.8 | 17.6 | 4.5 | 8.0 | 3.3 | 8.5 | 3.3 | 11.6 | 3.4 | 14.0 | 4.0 | 36.3 | 5.7 |
| **144** | 11.4 | 4.7 | 18.3 | 5.4 | 7.8 | 4.0 | 8.9 | 4.2 | 12.1 | 4.1 | 15.2 | 5.0 | 35.9 | 6.5 |
| **145** | 10.1 | 3.5 | 17.3 | 4.4 | 6.9 | 2.9 | 7.7 | 3.0 | 10.2 | 3.1 | 13.6 | 3.8 | 37.6 | 5.9 |
| **146** | 8.3 | 4.9 | 14.8 | 5.8 | 5.8 | 4.2 | 6.0 | 4.2 | 10.1 | 4.5 | 11.4 | 5.2 | 29.6 | 7.4 |
| **147** | 11.2 | 4.6 | 18.2 | 5.1 | 8.1 | 3.9 | 8.8 | 4.0 | 12.8 | 4.1 | 14.8 | 4.8 | 35.8 | 6.1 |
| **148** | 7.1 | 3.6 | 13.2 | 4.5 | 4.0 | 2.6 | 4.9 | 2.9 | 8.3 | 3.4 | 10.5 | 4.1 | 29.6 | 6.3 |
| **149** | 7.8 | 2.5 | 13.8 | 3.1 | 4.6 | 1.9 | 5.8 | 2.1 | 8.8 | 2.3 | 11.3 | 2.9 | 31.8 | 4.4 |
| **150** | 6.8 | 4.2 | 13.3 | 5.4 | 4.4 | 3.4 | 4.8 | 3.5 | 8.3 | 3.9 | 10.0 | 4.7 | 30.2 | 7.5 |
| **151** | 9.5 | 3.2 | 15.9 | 3.7 | 6.4 | 2.6 | 7.3 | 2.8 | 11.1 | 3.0 | 12.9 | 3.4 | 33.5 | 4.8 |
| **152** | 8.5 | 2.7 | 14.2 | 3.3 | 5.6 | 2.2 | 6.8 | 2.4 | 9.3 | 2.6 | 11.4 | 3.0 | 32.1 | 4.8 |
| **153** | 6.7 | 3.0 | 12.6 | 3.9 | 4.5 | 2.4 | 5.0 | 2.5 | 7.7 | 2.7 | 9.5 | 3.4 | 29.6 | 5.7 |
| **154** | 14.1 | 2.1 | 19.8 | 2.3 | 10.3 | 2.0 | 12.0 | 2.0 | 14.7 | 2.0 | 17.5 | 2.2 | 38.6 | 3.0 |
| **155** | 13.5 | 2.9 | 15.3 | 3.1 | 8.3 | 2.2 | 11.6 | 2.7 | 10.7 | 2.5 | 14.0 | 3.0 | 23.5 | 3.8 |
| **156** | 13.0 | 2.7 | 15.0 | 2.8 | 10.0 | 2.4 | 12.0 | 2.6 | 12.7 | 2.6 | 13.9 | 2.8 | 23.9 | 3.7 |
| **157** | 12.1 | 3.0 | 14.0 | 3.0 | 6.8 | 2.0 | 10.1 | 2.9 | 12.2 | 2.9 | 14.0 | 3.2 | 21.1 | 3.8 |
| **158** | 11.8 | 2.4 | 13.2 | 2.5 | 7.3 | 1.8 | 9.6 | 2.2 | 10.4 | 2.2 | 12.2 | 2.4 | 18.8 | 2.9 |
| **159** | 15.9 | 3.7 | 16.7 | 3.5 | 8.9 | 2.7 | 13.5 | 3.5 | 16.3 | 3.7 | 17.9 | 4.0 | 22.6 | 4.0 |
| **160** | 17.6 | 3.7 | 17.5 | 3.6 | 10.6 | 3.0 | 15.5 | 3.7 | 16.0 | 3.6 | 18.6 | 4.0 | 22.8 | 4.0 |
| **161** | 21.5 | 4.9 | 20.7 | 4.5 | 13.2 | 4.0 | 19.0 | 4.9 | 20.4 | 4.7 | 22.3 | 5.0 | 26.0 | 4.7 |
| **162** | 15.8 | 4.0 | 15.2 | 3.9 | 9.4 | 3.2 | 13.9 | 4.0 | 11.9 | 3.5 | 15.4 | 4.0 | 19.2 | 4.2 |
| **163** | 16.9 | 3.8 | 17.9 | 3.6 | 8.9 | 2.8 | 14.2 | 3.6 | 16.9 | 3.7 | 19.5 | 4.2 | 24.7 | 4.2 |
| **164** | 10.7 | 1.9 | 16.3 | 2.4 | 5.0 | 1.3 | 7.7 | 1.7 | 10.9 | 2.0 | 14.9 | 2.2 | 35.7 | 3.2 |
| **165** | 9.0 | 2.3 | 14.8 | 2.9 | 5.8 | 1.8 | 7.2 | 2.0 | 9.2 | 2.1 | 12.2 | 2.6 | 33.6 | 4.0 |
| **166** | 9.8 | 2.5 | 16.1 | 3.0 | 6.9 | 2.1 | 8.1 | 2.2 | 10.1 | 2.3 | 12.9 | 2.8 | 36.6 | 4.0 |
| **167** | 9.2 | 2.2 | 15.0 | 2.6 | 6.1 | 1.8 | 7.4 | 1.9 | 9.7 | 2.0 | 12.3 | 2.4 | 34.6 | 3.6 |
| **168** | 8.4 | 2.7 | 13.9 | 3.4 | 4.2 | 1.7 | 6.1 | 2.2 | 7.9 | 2.4 | 12.1 | 3.3 | 31.8 | 5.1 |
| **169** | 8.2 | 2.2 | 13.6 | 2.7 | 4.4 | 1.5 | 6.1 | 1.8 | 8.4 | 2.0 | 11.7 | 2.6 | 30.9 | 4.1 |
| **170** | 9.5 | 1.7 | 14.0 | 2.1 | 6.0 | 1.4 | 7.7 | 1.6 | 10.4 | 1.7 | 12.1 | 1.9 | 29.3 | 3.1 |
| **171** | 10.8 | 4.9 | 13.4 | 5.2 | 10.5 | 5.1 | 11.4 | 5.3 | 12.3 | 5.1 | 15.6 | 5.5 | 24.8 | 6.3 |
| **172** | 4.8 | 2.4 | 8.4 | 3.3 | 3.0 | 1.9 | 3.6 | 2.1 | 4.5 | 1.9 | 6.1 | 2.6 | 17.0 | 5.0 |
| **173** | 4.7 | 2.4 | 8.7 | 3.3 | 2.8 | 1.7 | 3.4 | 2.0 | 4.6 | 2.0 | 6.5 | 2.7 | 19.2 | 5.2 |
| **174** | 7.0 | 2.1 | 10.9 | 2.7 | 3.3 | 1.3 | 5.1 | 1.8 | 6.9 | 2.0 | 9.7 | 2.6 | 22.1 | 4.2 |
| **175** | 6.2 | 1.8 | 10.3 | 2.3 | 3.5 | 1.3 | 4.6 | 1.6 | 7.4 | 1.9 | 8.6 | 2.2 | 21.8 | 3.7 |
| **176** | 5.0 | 2.6 | 9.2 | 3.6 | 2.9 | 1.9 | 3.6 | 2.1 | 4.9 | 2.1 | 6.9 | 3.0 | 20.8 | 5.7 |
| **177** | 6.4 | 2.4 | 10.9 | 3.2 | 3.1 | 1.6 | 4.6 | 2.0 | 6.3 | 2.2 | 9.2 | 2.9 | 24.5 | 5.1 |
| **178** | 6.4 | 2.4 | 11.1 | 3.3 | 3.3 | 1.6 | 4.7 | 2.0 | 6.0 | 2.1 | 9.2 | 3.0 | 25.7 | 5.3 |
| **179** | 13.1 | 3.2 | 14.5 | 3.3 | 9.8 | 2.9 | 11.9 | 3.2 | 11.3 | 2.9 | 13.2 | 3.1 | 21.3 | 3.8 |
| **180** | 14.4 | 2.4 | 19.5 | 2.6 | 9.9 | 2.0 | 11.3 | 2.1 | 14.1 | 2.3 | 16.5 | 2.5 | 34.0 | 3.2 |
| **181** | 19.4 | 3.4 | 21.2 | 3.4 | 14.5 | 3.2 | 16.6 | 3.3 | 17.0 | 3.4 | 19.2 | 3.4 | 28.3 | 3.8 |
| **182** | 6.5 | 9.9 | 8.8 | 9.0 | 5.3 | 9.4 | 4.8 | 8.9 | 11.5 | 12.3 | 7.5 | 9.6 | 8.5 | 7.5 |
| **183** | 6.7 | 9.7 | 9.5 | 9.2 | 5.2 | 9.1 | 4.9 | 8.8 | 11.1 | 11.5 | 8.0 | 9.6 | 10.2 | 8.2 |
| **184** | 7.6 | 7.7 | 10.6 | 7.3 | 6.2 | 7.2 | 5.8 | 6.9 | 12.0 | 9.1 | 8.8 | 7.5 | 12.9 | 7.2 |
| **185** | 8.3 | 6.6 | 11.1 | 6.2 | 6.7 | 6.1 | 6.5 | 6.1 | 12.1 | 7.3 | 9.5 | 6.4 | 13.8 | 6.2 |
| **186** | 6.4 | 10.6 | 9.4 | 10.3 | 5.0 | 9.9 | 4.5 | 9.4 | 11.2 | 13.0 | 7.8 | 10.6 | 10.3 | 9.5 |
| **187** | 7.0 | 7.5 | 9.8 | 7.1 | 5.7 | 7.0 | 5.2 | 6.6 | 11.7 | 9.1 | 8.3 | 7.4 | 11.5 | 6.8 |
| **188** | 7.4 | 6.7 | 10.6 | 6.6 | 6.0 | 6.3 | 5.6 | 5.9 | 10.7 | 7.3 | 8.7 | 6.5 | 13.2 | 6.4 |
| **189** | 15.7 | 3.8 | 16.3 | 3.7 | 11.9 | 3.5 | 14.6 | 3.8 | 14.5 | 3.6 | 16.4 | 3.8 | 22.1 | 4.2 |
| **190** | 6.0 | 9.5 | 8.5 | 9.0 | 4.5 | 8.7 | 4.2 | 8.4 | 10.1 | 11.5 | 7.1 | 9.4 | 8.7 | 7.8 |
| **191** | 6.0 | 8.4 | 8.3 | 7.7 | 4.7 | 7.7 | 4.4 | 7.5 | 10.0 | 10.0 | 6.9 | 8.1 | 8.6 | 6.7 |
| **192** | 6.5 | 6.2 | 8.4 | 5.6 | 4.8 | 5.3 | 5.0 | 5.7 | 9.6 | 7.0 | 7.3 | 6.0 | 9.1 | 5.2 |
| **193** | 8.2 | 3.2 | 9.9 | 3.1 | 5.4 | 2.5 | 6.6 | 3.0 | 8.8 | 3.0 | 9.0 | 3.2 | 13.2 | 3.4 |
| **194** | 8.2 | 4.0 | 10.5 | 3.8 | 5.7 | 3.2 | 6.5 | 3.7 | 9.7 | 3.8 | 9.5 | 3.9 | 14.2 | 4.1 |
| **195** | 13.6 | 3.2 | 14.3 | 3.2 | 8.0 | 2.5 | 11.4 | 3.0 | 11.1 | 2.9 | 14.1 | 3.3 | 20.2 | 3.7 |
| **196** | 11.5 | 3.1 | 12.2 | 3.0 | 8.4 | 2.7 | 10.5 | 3.0 | 10.6 | 2.8 | 11.5 | 3.0 | 16.3 | 3.4 |
| **197** | 11.4 | 2.8 | 12.5 | 2.9 | 7.4 | 2.3 | 9.9 | 2.7 | 9.7 | 2.5 | 11.7 | 2.8 | 17.1 | 3.3 |
| **198** | 12.3 | 3.3 | 13.7 | 3.3 | 9.9 | 3.1 | 11.4 | 3.2 | 11.3 | 3.0 | 12.5 | 3.2 | 19.4 | 3.7 |
| **199** | 13.6 | 3.6 | 15.1 | 3.6 | 10.8 | 3.3 | 12.6 | 3.5 | 11.8 | 3.2 | 13.8 | 3.4 | 21.3 | 4.1 |
| **200** | 17.0 | 3.9 | 17.6 | 3.8 | 12.1 | 3.5 | 15.3 | 3.8 | 15.2 | 3.6 | 17.2 | 3.8 | 23.5 | 4.2 |
| **201** | 11.5 | 5.3 | 12.9 | 5.2 | 11.4 | 5.8 | 12.7 | 5.8 | 14.0 | 5.7 | 15.3 | 5.6 | 22.6 | 6.0 |
| **202** | 8.2 | 3.9 | 10.4 | 4.1 | 7.5 | 4.0 | 8.0 | 4.0 | 10.6 | 4.5 | 11.1 | 4.3 | 16.9 | 4.6 |
| **203** | 8.2 | 5.3 | 10.5 | 5.1 | 6.3 | 4.9 | 6.5 | 5.0 | 12.4 | 6.6 | 10.0 | 5.7 | 14.2 | 5.6 |
| **204** | 13.6 | 4.5 | 14.0 | 4.4 | 7.8 | 3.4 | 10.9 | 4.0 | 9.0 | 3.6 | 12.6 | 4.2 | 15.5 | 4.3 |
| **205** | 13.2 | 4.2 | 14.6 | 4.3 | 6.6 | 2.9 | 10.7 | 3.8 | 10.1 | 3.6 | 14.7 | 4.6 | 19.1 | 4.8 |
| **206** | 12.2 | 3.8 | 14.6 | 4.0 | 5.9 | 2.6 | 9.7 | 3.4 | 10.2 | 3.4 | 14.7 | 4.4 | 21.1 | 4.9 |
| **207** | 10.8 | 3.4 | 13.4 | 3.7 | 5.6 | 2.3 | 7.9 | 2.8 | 7.5 | 2.7 | 11.3 | 3.3 | 19.0 | 4.4 |
| **208** | 11.2 | 3.6 | 13.0 | 3.8 | 5.8 | 2.4 | 8.6 | 3.0 | 7.3 | 2.7 | 11.2 | 3.5 | 18.1 | 4.5 |
| **209** | 10.3 | 2.3 | 12.2 | 2.5 | 5.5 | 1.6 | 7.9 | 2.1 | 9.0 | 2.2 | 11.3 | 2.5 | 18.7 | 3.0 |
| **210** | 10.9 | 2.4 | 12.5 | 2.6 | 5.6 | 1.6 | 8.2 | 2.1 | 9.0 | 2.2 | 11.4 | 2.5 | 18.1 | 3.0 |
| **211** | 14.1 | 4.2 | 16.2 | 4.3 | 7.1 | 3.0 | 11.7 | 3.9 | 11.9 | 3.8 | 17.0 | 4.9 | 23.8 | 5.3 |
| **212** | 14.3 | 3.7 | 14.6 | 3.7 | 8.9 | 3.1 | 11.9 | 3.5 | 13.1 | 3.7 | 14.3 | 3.7 | 20.5 | 4.0 |
| **213** | 11.3 | 4.9 | 12.1 | 4.8 | 12.2 | 5.8 | 12.5 | 5.6 | 13.6 | 5.6 | 14.3 | 5.4 | 17.7 | 5.2 |
| **214** | 12.7 | 2.6 | 20.2 | 3.1 | 7.3 | 1.9 | 9.9 | 2.4 | 14.2 | 2.8 | 18.2 | 3.0 | 44.9 | 3.8 |
| **215** | 13.3 | 2.3 | 19.6 | 2.8 | 6.8 | 1.6 | 10.1 | 2.2 | 14.3 | 2.5 | 18.0 | 2.6 | 41.8 | 3.5 |
| **216** | 13.4 | 2.7 | 20.5 | 3.1 | 7.1 | 1.9 | 10.4 | 2.4 | 13.9 | 2.7 | 18.5 | 3.0 | 45.0 | 4.0 |
| **217** | 14.0 | 3.0 | 20.9 | 3.5 | 6.5 | 1.9 | 10.5 | 2.7 | 15.5 | 3.2 | 20.0 | 3.5 | 44.4 | 4.4 |
| **218** | 12.9 | 2.5 | 18.9 | 3.0 | 5.6 | 1.6 | 9.4 | 2.3 | 13.6 | 2.7 | 17.8 | 2.9 | 40.4 | 3.9 |
| **219** | 14.4 | 3.6 | 21.3 | 4.2 | 5.9 | 2.1 | 10.6 | 3.2 | 14.2 | 3.5 | 20.8 | 4.3 | 45.7 | 5.3 |
| **220** | 9.6 | 2.3 | 13.8 | 2.9 | 4.0 | 1.4 | 6.4 | 1.8 | 7.6 | 2.0 | 11.2 | 2.5 | 27.7 | 3.8 |
| **221** | 10.1 | 1.7 | 15.8 | 2.2 | 7.2 | 1.5 | 8.0 | 1.5 | 10.5 | 1.7 | 12.6 | 1.9 | 33.1 | 3.1 |
| **222** | 11.0 | 1.9 | 16.7 | 2.3 | 8.1 | 1.8 | 9.0 | 1.7 | 11.0 | 1.8 | 13.4 | 2.0 | 34.7 | 3.1 |
| **223** | 11.4 | 1.6 | 17.0 | 2.0 | 8.0 | 1.5 | 9.2 | 1.4 | 12.2 | 1.6 | 14.2 | 1.8 | 34.7 | 2.7 |
| **224** | 12.8 | 1.6 | 18.5 | 2.0 | 7.7 | 1.3 | 9.9 | 1.5 | 13.3 | 1.7 | 16.1 | 1.8 | 36.5 | 2.5 |
| **225** | 13.6 | 1.8 | 18.4 | 2.0 | 9.3 | 1.6 | 11.2 | 1.7 | 13.9 | 1.8 | 16.4 | 2.0 | 33.3 | 2.5 |
| **226** | 11.6 | 2.6 | 17.7 | 3.1 | 8.5 | 2.3 | 9.9 | 2.4 | 11.1 | 2.3 | 14.6 | 2.8 | 38.4 | 4.1 |
| **227** | 13.8 | 1.8 | 19.7 | 2.2 | 8.4 | 1.5 | 10.4 | 1.6 | 13.1 | 1.9 | 16.6 | 1.9 | 38.2 | 2.7 |
| **228** | 16.2 | 2.3 | 22.1 | 2.7 | 10.3 | 1.9 | 12.7 | 2.1 | 15.5 | 2.3 | 18.3 | 2.5 | 40.2 | 3.2 |
| **229** | 13.5 | 2.0 | 19.5 | 2.3 | 10.3 | 1.9 | 11.3 | 1.8 | 13.8 | 1.9 | 16.1 | 2.0 | 37.6 | 2.8 |
| **230** | 13.9 | 1.7 | 19.9 | 2.1 | 9.5 | 1.6 | 11.1 | 1.6 | 14.1 | 1.7 | 16.6 | 1.8 | 38.9 | 2.5 |
| **231** | 13.1 | 1.8 | 18.6 | 2.1 | 9.5 | 1.7 | 10.7 | 1.6 | 13.8 | 1.8 | 15.6 | 1.9 | 36.5 | 2.7 |
| **232** | 20.0 | 3.4 | 27.0 | 3.8 | 14.4 | 3.2 | 16.8 | 3.2 | 22.3 | 3.5 | 24.9 | 3.9 | 45.1 | 4.1 |
| **233** | 14.1 | 2.7 | 20.8 | 3.2 | 10.5 | 2.4 | 11.5 | 2.4 | 14.5 | 2.7 | 17.5 | 3.0 | 39.4 | 4.0 |
| **234** | 35.6 | 5.4 | 40.8 | 5.5 | 25.8 | 5.0 | 31.9 | 5.5 | 30.9 | 5.1 | 37.0 | 5.4 | 58.3 | 5.1 |
| **235** | 34.7 | 5.7 | 40.2 | 5.7 | 24.4 | 5.0 | 30.8 | 5.6 | 29.7 | 5.3 | 36.4 | 5.6 | 58.7 | 5.3 |
| **236** | 34.2 | 4.5 | 40.1 | 4.6 | 25.6 | 4.0 | 30.5 | 4.5 | 31.4 | 4.3 | 35.9 | 4.4 | 58.7 | 4.4 |
| **237** | 37.4 | 4.7 | 42.0 | 4.7 | 28.8 | 4.3 | 33.7 | 4.7 | 34.5 | 4.5 | 38.3 | 4.6 | 58.2 | 4.5 |
| **238** | 34.5 | 4.7 | 39.5 | 4.7 | 25.7 | 4.2 | 30.8 | 4.7 | 31.9 | 4.5 | 35.8 | 4.6 | 56.3 | 4.6 |
| **239** | 34.3 | 4.2 | 40.5 | 4.0 | 27.1 | 3.8 | 30.3 | 4.0 | 34.3 | 4.0 | 36.3 | 3.9 | 58.3 | 4.0 |
| **240** | 36.7 | 4.6 | 42.2 | 4.5 | 28.7 | 4.1 | 33.1 | 4.5 | 35.1 | 4.3 | 38.4 | 4.3 | 59.4 | 4.3 |
| **241** | 33.7 | 4.7 | 39.0 | 4.7 | 25.1 | 4.1 | 29.9 | 4.7 | 31.7 | 4.5 | 35.2 | 4.5 | 56.4 | 4.7 |
| **242** | 32.0 | 3.9 | 37.4 | 4.0 | 22.8 | 3.4 | 27.7 | 3.8 | 28.6 | 3.7 | 33.6 | 3.8 | 54.1 | 3.9 |
| **243** | 33.3 | 4.3 | 38.7 | 4.4 | 24.5 | 3.8 | 29.4 | 4.2 | 30.9 | 4.1 | 34.9 | 4.2 | 56.0 | 4.3 |
| **244** | 36.8 | 4.9 | 39.9 | 4.9 | 28.8 | 4.7 | 33.5 | 5.0 | 34.9 | 4.7 | 37.2 | 4.8 | 52.7 | 4.7 |
| **245** | 34.5 | 4.0 | 39.8 | 4.1 | 25.9 | 3.6 | 30.6 | 4.0 | 31.4 | 3.8 | 35.9 | 3.9 | 57.1 | 3.9 |
| **246** | 36.1 | 5.3 | 39.1 | 5.2 | 27.9 | 4.9 | 33.0 | 5.5 | 33.3 | 5.1 | 35.9 | 5.1 | 52.7 | 5.2 |
| **247** | 30.1 | 3.0 | 38.8 | 3.2 | 22.6 | 2.8 | 25.2 | 2.7 | 28.5 | 2.9 | 33.2 | 3.0 | 59.8 | 3.2 |
| **248** | 29.3 | 4.0 | 38.2 | 4.1 | 19.9 | 3.5 | 24.1 | 3.7 | 25.2 | 3.7 | 32.7 | 3.9 | 61.6 | 3.9 |
| **249** | 32.7 | 4.0 | 39.5 | 4.3 | 25.7 | 4.0 | 27.7 | 4.0 | 31.6 | 4.1 | 35.1 | 4.2 | 59.3 | 4.3 |
| **250** | 41.2 | 6.6 | 50.9 | 6.8 | 34.1 | 6.7 | 35.3 | 6.5 | 40.1 | 6.9 | 45.5 | 6.8 | 71.0 | 5.6 |
| **251** | 41.8 | 7.2 | 51.2 | 7.2 | 35.8 | 7.4 | 36.4 | 7.1 | 42.0 | 7.5 | 46.4 | 7.3 | 70.3 | 6.0 |
| **252** | 40.1 | 5.2 | 48.5 | 5.1 | 31.5 | 4.9 | 35.3 | 5.0 | 40.1 | 5.1 | 43.6 | 4.9 | 71.0 | 4.2 |
| **253** | 36.6 | 4.2 | 45.4 | 4.5 | 28.7 | 4.1 | 31.0 | 3.9 | 36.9 | 4.4 | 40.0 | 4.3 | 67.6 | 3.9 |
| **254** | 35.7 | 4.3 | 46.1 | 4.3 | 27.4 | 4.0 | 30.5 | 4.0 | 35.0 | 4.2 | 40.2 | 4.1 | 70.0 | 3.6 |
| **255** | 36.1 | 4.5 | 46.6 | 4.5 | 27.4 | 4.2 | 30.8 | 4.2 | 35.2 | 4.4 | 40.7 | 4.4 | 71.1 | 3.7 |
| **256** | 37.3 | 5.1 | 47.0 | 5.0 | 29.4 | 4.8 | 32.7 | 4.9 | 38.0 | 5.1 | 41.5 | 4.8 | 70.6 | 4.1 |
| **257** | 42.6 | 6.7 | 51.7 | 6.7 | 35.6 | 6.8 | 36.8 | 6.7 | 42.3 | 6.8 | 46.8 | 6.9 | 71.6 | 5.5 |
| **258** | 42.4 | 6.6 | 49.1 | 6.6 | 34.7 | 6.7 | 37.7 | 6.6 | 38.8 | 6.6 | 45.3 | 6.7 | 65.9 | 5.9 |
| **259** | 33.2 | 4.2 | 43.3 | 4.3 | 24.2 | 3.8 | 28.1 | 3.9 | 31.6 | 4.1 | 37.6 | 4.1 | 67.9 | 3.7 |
