# Supplementary material for: Towards soil-transmitted helminths transmission interruption: The impact of diagnostic tools on infection prediction in a low intensity setting in Southern Mozambique
Source: PLoS Negl Trop Dis. 2021 Oct 25;15(10):e0009803. doi: 10.1371/journal.pntd.0009803 (PMC8568186; doi:10.1371/journal.pntd.0009803)
Supplement: S1 Fig — A) Distribution of all households (black dots) in Manhiça district. B) Household location of study participants (black dots) after regular sampling. Base layer map obtained in https://gadm.org/download_country_v3.html (DOCX) [file pntd.0009803.s005.docx]

S1 Fig. Selection of study participants was performed using regular sampling design. A) Distribution of Manhiça district households (blue dots). B) Division of Manhiça district households (blue dots) in a regular grid (1,750 m x 1,750m). C) Location of centroid (red dots) in each cell of the grid. D) Location of all Manhiça district households (black dots) in Manhiça district (white perimeter area). E) Location of study participants’ households (black dots) in Manhiça district (white perimeter area) after conducting regular sampling. Base layer map obtained in https://data.humdata.org/dataset/mozambique-administrative-levels-0-3

**A B C**


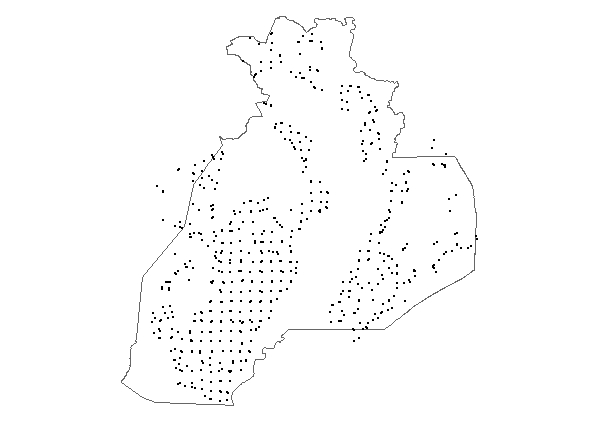
**
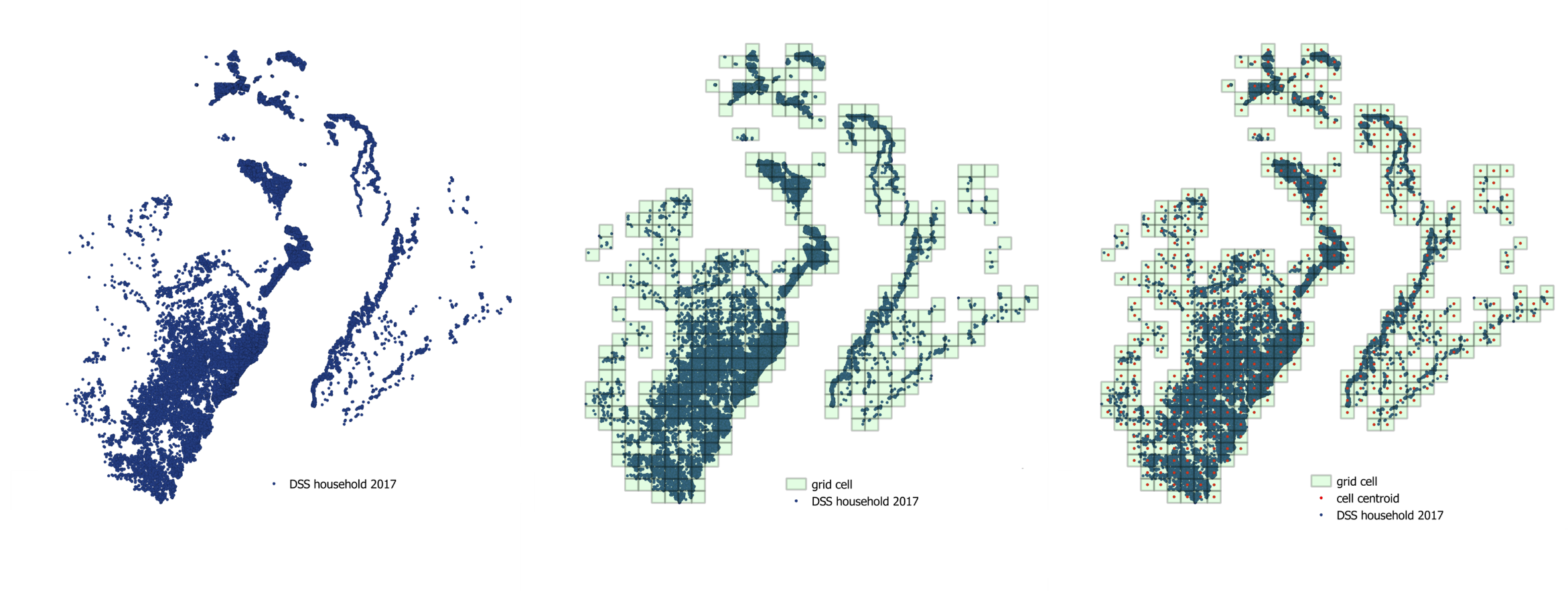
**
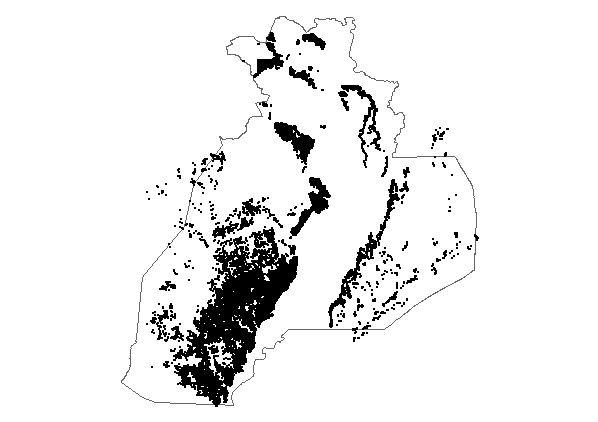
**D E**
