# Supplementary material for: Towards soil-transmitted helminths transmission interruption: The impact of diagnostic tools on infection prediction in a low intensity setting in Southern Mozambique
Source: PLoS Negl Trop Dis. 2021 Oct 25;15(10):e0009803. doi: 10.1371/journal.pntd.0009803 (PMC8568186; doi:10.1371/journal.pntd.0009803)

S2 Fig. Assessment of spatial autocorrelation in the residuals. Telemann from one and two stools, single Kato-Katz from one or two stool samples, duplicate Kato-Katz from one or two stools and qPCR variograms after fitting the binomial generalized linear model of at least one STH infection.


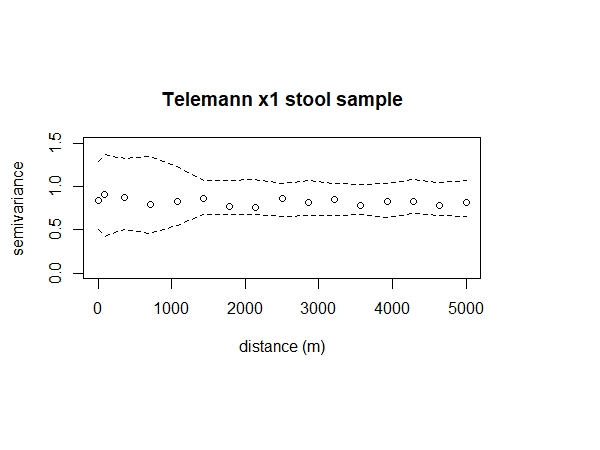

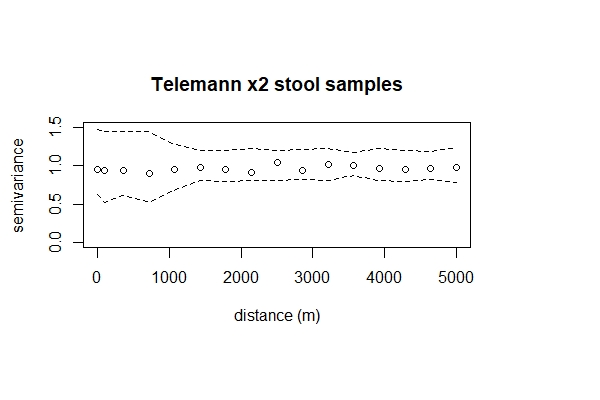

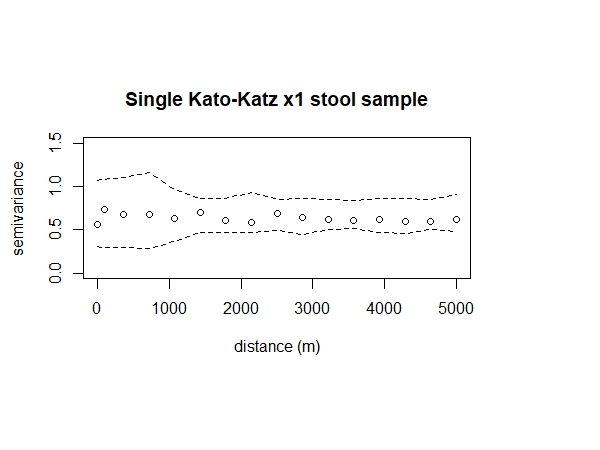


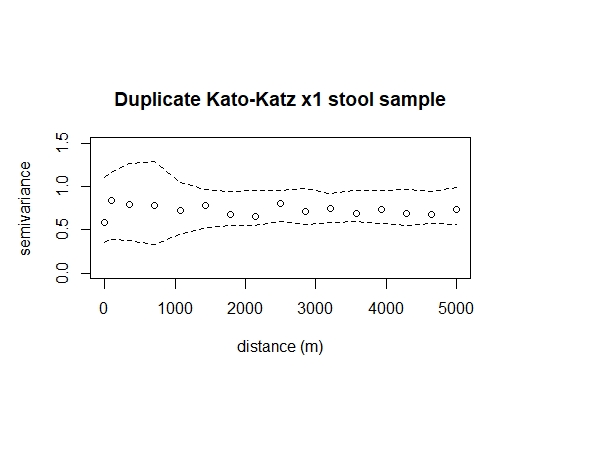


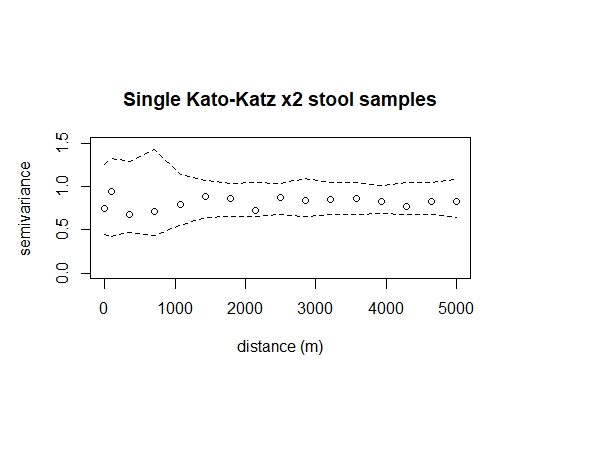


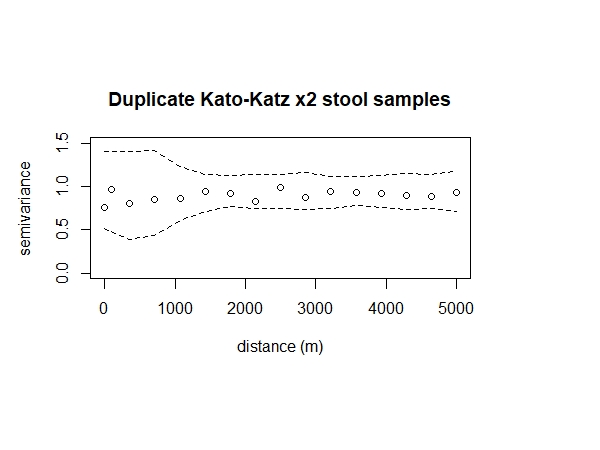


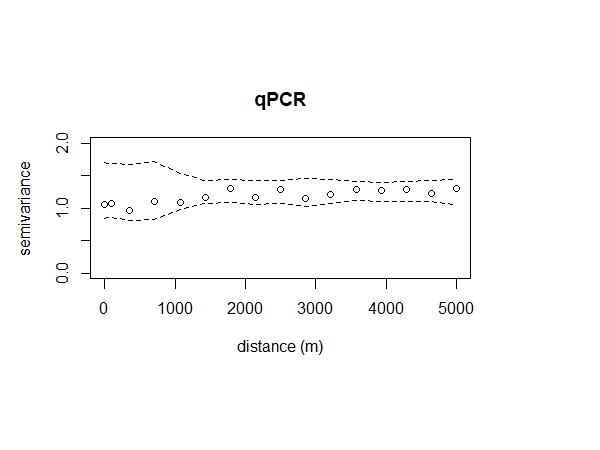

Supplement: S2 Fig — Telemann from one or two stools, single Kato-Katz from one or two stool samples, duplicate Kato-Katz from one or two stools and qPCR variograms after fitting the binomial generalized linear model of at least one STH infection. (DOCX) [file pntd.0009803.s006.docx]
