# Supplementary material for: Towards soil-transmitted helminths transmission interruption: The impact of diagnostic tools on infection prediction in a low intensity setting in Southern Mozambique
Source: PLoS Negl Trop Dis. 2021 Oct 25;15(10):e0009803. doi: 10.1371/journal.pntd.0009803 (PMC8568186; doi:10.1371/journal.pntd.0009803)

S3 Fig. Faecal egg count agreement of single Kato-Katz in one stool with the other quantitative microscopic methods (duplicate Kato-Katz in one stool sample, and single and duplicate Kato-Katz in two stool samples) for *A. lumbricoides*, *T. trichiura* and hookworm. In each graph, the concordance correlation coefficient (ρ) and p-value are provided. The dashed line corresponds to the perfect correlation.


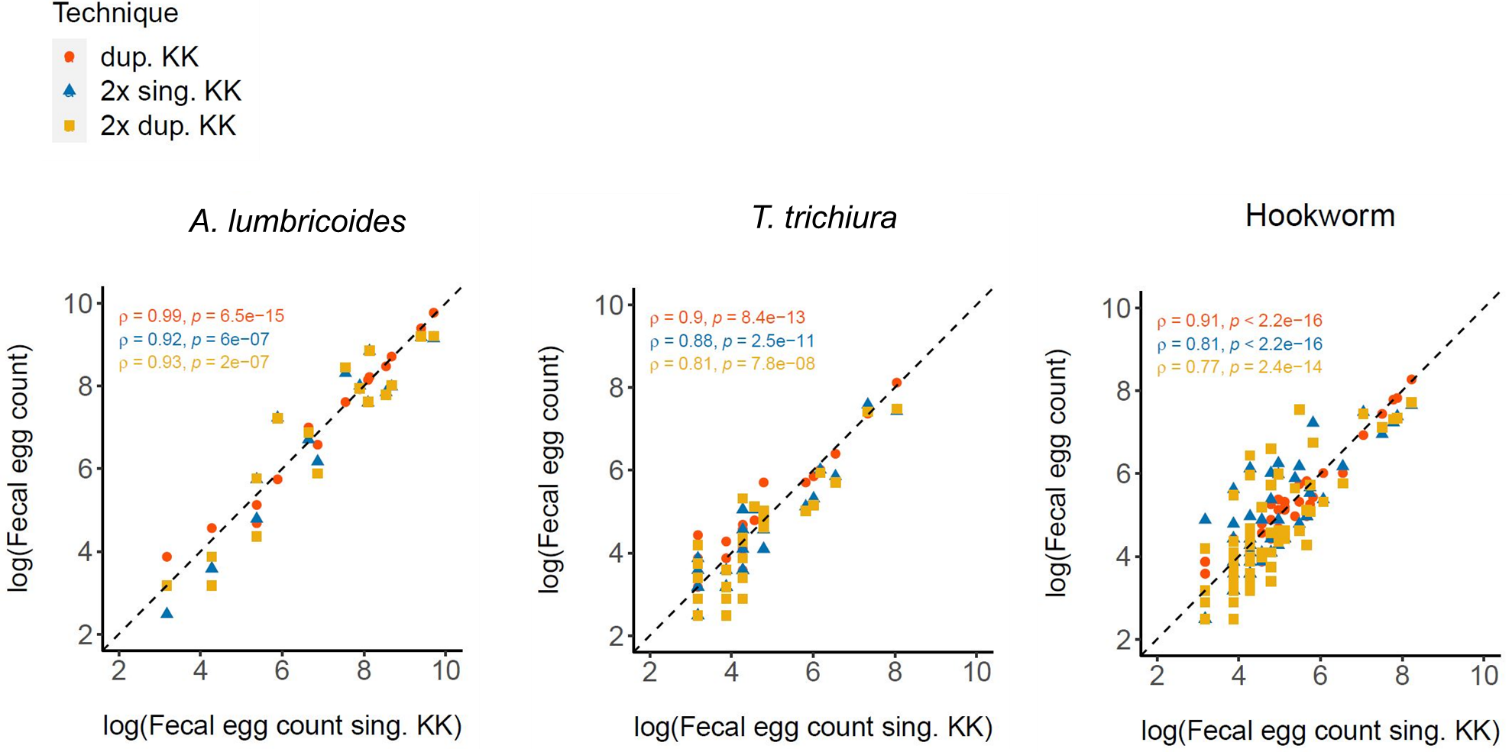

Supplement: S3 Fig — In each graph, the concordance correlation coefficient (ρ) and p-value are provided. The dashed line corresponds to the perfect correlation. (DOCX) [file pntd.0009803.s007.docx]
