# Supplementary material for: Towards soil-transmitted helminths transmission interruption: The impact of diagnostic tools on infection prediction in a low intensity setting in Southern Mozambique
Source: PLoS Negl Trop Dis. 2021 Oct 25;15(10):e0009803. doi: 10.1371/journal.pntd.0009803 (PMC8568186; doi:10.1371/journal.pntd.0009803)

**S4 Fig. District map of the probability of the estimated prevalence of at least one STH infection exceeding 20% (WHO threshold to start MDA). It was calculated with a generalized linear model assuming a binomial distribution for Telemann in one and two stools, single and duplicate Kato-Katz in one stool, single and duplicate Kato-Katz in two stools and qPCR in one stool. Base layer map obtained in https://data.humdata.org/dataset/mozambique-administrative-levels-0-3**


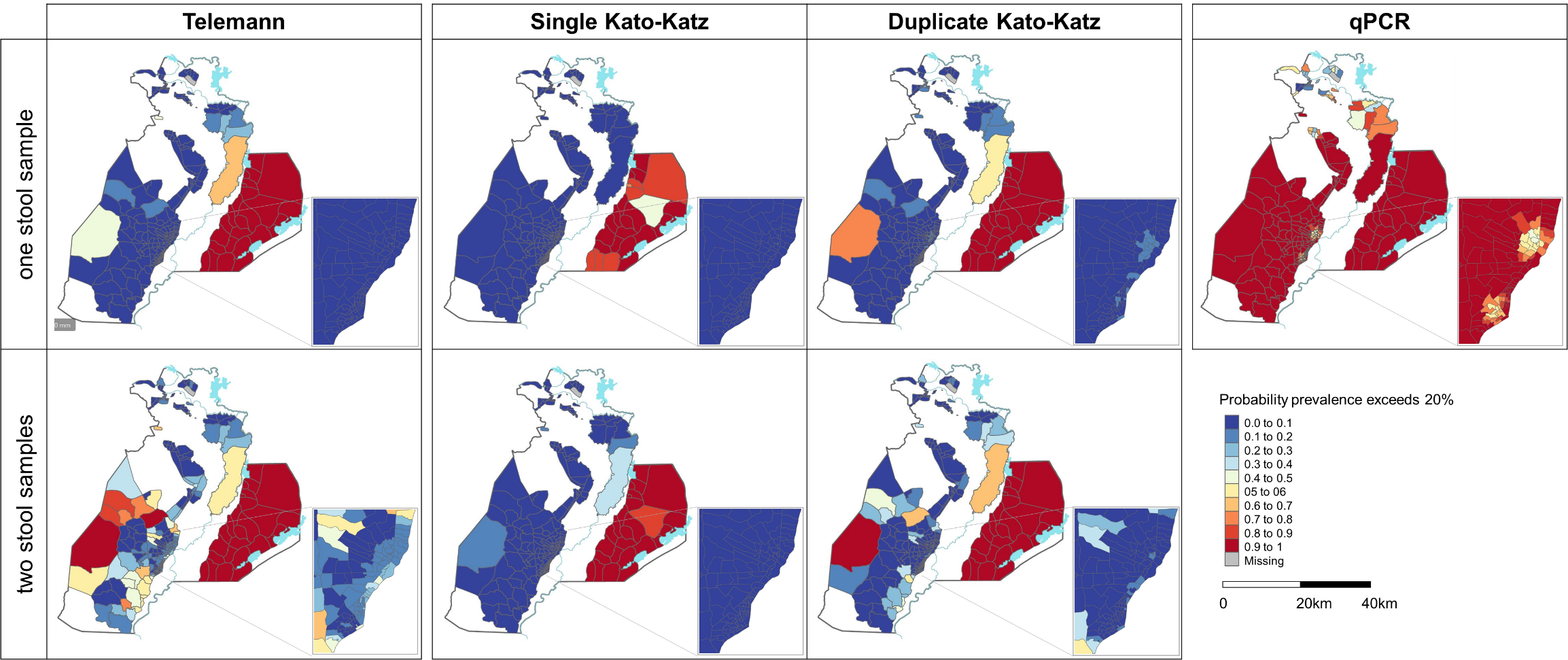

Supplement: S4 Fig — It was calculated with a generalized linear model assuming a binomial distribution for Telemann in one and two stools, single and duplicate Kato-Katz in one stool, single and duplicate Kato-Katz in two stools and qPCR in one stool. Base layer map obtained in https://gadm.org/download_country_v3.html (DOCX) [file pntd.0009803.s008.docx]
