# Supplementary material for: Towards soil-transmitted helminths transmission interruption: The impact of diagnostic tools on infection prediction in a low intensity setting in Southern Mozambique
Source: PLoS Negl Trop Dis. 2021 Oct 25;15(10):e0009803. doi: 10.1371/journal.pntd.0009803 (PMC8568186; doi:10.1371/journal.pntd.0009803)
Supplement: Attachment — Submitted filename: Comments.docx [file pntd.0009803.s009.docx]

Introduction

This study is important as it has evaluated three diagnostic techniques to detect STH infection in a low intensity setting in Southern Mozambique and estimated STH prevalence to identify the locations with ongoing transmission. However, there are a number of issues which require addressing.

Comments;

1. Line 89: please mention/replace what is meant by “they” in the sentence.
2. Lines 91 – 92 : sentence is not clear, please reword the sentence.
3. Give a reference for the sentence in line 92 – 93 (i.e. rebound infection)
4. Line 130: please explain in detail what is meant by regular sampling design with respect to the current study design (i.e. providing references are not adequate here).
   - Explain whether there is an original survey, if so, explain it’s sampling units, sample size ect. where you in detail.
   - Is this s secondary data analysis? Did you take a sample from a survey? If so, explain why did you select a sample for this study.
   - Descried S1 figure in detail.
5. Lines 131 - 132 : is the sentence correct (1,750km by 1,750km resolution)?
   - What is the total area (km^2^) in the study region? Please include.
6. Lines 140 – 144 : describe how did you distribute 384 over the study region (what is the relationship between 384 people and 440 square areas?)
7. Line 222 : explain the socioeconomic variable you considered in the study and how SES was developed.
8. Line 254 : provide a reference for considering correlation coefficient of 0.8 as the cut off
9. Lines 270 – 272: explain why did you fit a negative binomial model in this study and provide details of the fitted model including considered covariates, assessment of non-linear associations in the covariates, model fit statistics, was there any over dispersion, if so did you consider generalized mixed effect model and assessed the necessity of random effects?
10. S3 Figure: Is the figure legend correct? Why did you fit a the binomial generalized linear model if there is no spatial correlation?
11. Line 273: explain the spatial units
12. Results section : authors have done significance testing and provide P values, but have not mentioned which test/s have been used, please include them under methods
13. Lines 302 – 303 : give a breakdown for < 15 and > 15
14. Lines 391 – 320 and 321 – 322 : evaluate the differences and provide p values
15. Line 372 : explain what is meant by fair correlation
16. Fig 3: Legend should be corrected as Log(Fecal egg count) agreement with qPCR Ct value…
17. Lines 383 – 384: previously authors mentioned that they used 0.8 as the cut off and here mentioned as 0.65, which is correct? (this can be presented in the methods, not under the results)
18. Line 386 – explain what is meant by all variograms and give details (again can include under the methods)
19. Figure 4 – what do the white colour areas represent in the map? I can’t see much grey colour regions even though it is mentioned.
    - Prevalence estimates can be associated with standard errors and how can you quantify the certainty in the prevalence estimates in these maps?
    - Suggest to develop maps to show the probability of exceeding/non-exceeding the given WHO thresholds in order to facilitate decision making as this is one of the main objectives of this paper.
20. Line 431 : explain how can you infer an increasing sensitivity tendency if the data does not support
21. Lines 433 – 442 : authors have brough up new points which are not mentioned previously. Suggest to include these section under results first and discuss.
22. Line 460: explain what Farrel et al 2018 did.
23. Lines 478 : explain nested sampling in the methods.
24. Lines 479 – 483: authors need to need to explain this in the methods in details and then discuss.
    - Anyway, what is meant by geospatial model here?
25. Lines 495 onwards: Authors mentioned that the geospatial prediction as an essential instrument for targeting STH infection areas and discusses the diagnostic implication in this prediction. However, this has not been adequately discussed under the discussion. Please expand this section.
