# Supplementary material for: Towards soil-transmitted helminths transmission interruption: The impact of diagnostic tools on infection prediction in a low intensity setting in Southern Mozambique
Source: PLoS Negl Trop Dis. 2021 Oct 25;15(10):e0009803. doi: 10.1371/journal.pntd.0009803 (PMC8568186; doi:10.1371/journal.pntd.0009803)
Supplement: Attachment — Submitted filename: Comments_PLOSNTDs_editor_answered_08072021.docx [file pntd.0009803.s010.docx]

**Editor's comments**

Introduction

This study is important as it has evaluated three diagnostic techniques to detect STH infection in a low intensity setting in Southern Mozambique and estimated STH prevalence to identify the locations with ongoing transmission. However, there are a number of issues which require addressing.

Comments;

1. ***Line 89: please mention/replace what is meant by “they” in the sentence.***

Answer: We replaced it by “WHO”, the World Health Organization.

2. ***Lines 91 – 92 : sentence is not clear, please reword the sentence.***

Answer: We rewrote the sentence to make it more clear. “Some regions are approaching WHO goal of reducing the proportion of moderate and high intensity infections to less than 2% and, thus, reaching STH elimination as a public health problem (4, 5).”

3. ***Give a reference for the sentence in line 92 – 93 (i.e. rebound infection)***

Answer: We provided a reference of a systematic review and meta-analysis on soil-transmitted helminth reinfection after drug treatment: Jia, T. W., et al. (2012). Soil-transmitted helminth reinfection after drug treatment: a systematic review and meta-analysis. PLoS Negl Trop Dis 6(5): e1621. This described that after either targeted or mass drug administration, STH infections prevalence recovered rapidly in most endemic areas, with the prevalence of STH to be at or above half the initial level 6 months post-treatment.

4. ***Line 130: please explain in detail what is meant by regular sampling design with respect to the current study design (i.e. providing references are not adequate here).***

Answer: We explained in detail what is meant by a regular sampling design. The explanation in the manuscript is the following: “A regular sampling design was used to obtain spatial representativeness. A regular sampling design is a uniform sampling methodology specified in advance of data collection that sampling points are selected relative to a sampling origin. (14, 15). To design it, we divided the whole study area using a regular grid formed by 440 square areas of 1,750km by 1,750km resolution. One household was selected by defined area, the closest to the centroid, and then one child between 5 to 15 years of age and one person older than 15 years old were randomly selected and invited to take part in the study. In case of participation declining, the following household closest to the centroid was selected and so forth.” S1 Figure illustrates the location of our sampling.

- ***Explain whether there is an original survey, if so, explain it’s sampling units, sample size ect. where you in detail.***

Answer: The regular sampling design was conducted specifically for this study in order to estimate STH infection in Manhiça district. It is explained in lines 132-143. We estimated a minimum of 384 participants per group necessary to identify STH prevalence in Manhiça district. Since two consecutive samples per participant were required, we added 10% of loss to follow up or participation rejection in all square area. We needed to divide equally our study area for regular sampling, thus, we divided our study area by areas of 1,750m by 1,750m resolution including at least a household inside. The division of the area increased our 10% of loss to follow-up to 13%. Thus, we required to recruit 440 participants younger than 15 years old and 440 participants older than 15 years old.

- ***Is this s secondary data analysis? Did you take a sample from a survey? If so, explain why did you select a sample for this study.***

Answer: No, it is not a secondary data analysis. We included in this study all participants with microscopy results from two stools (Telemann and Kato-Katz techniques), qPCR results from one stool and demographic data from CISM DSS necessary for statistical modelling of infection (n = 792).

- ***Descried S1 figure in detail.***

Answer: S1 figure displays the location of all households in Manhiça district (A) and the location of study participants’ households (B). We modified S1 figure legend for: “S1 Figure. A) Location of all households (black dots) in Manhiça district (white perimeter area). B) Location of study participants’ households (black dots) in Manhiça district (white perimeter area). Study participants were selected using a regular sampling design.”

5. ***Lines 131 - 132 : is the sentence correct (1,750km by 1,750km resolution)?***
   - ***What is the total area (km^2^) in the study region? Please include.***

Answer: We agree with the editor that there was a mistake on “1,750km by 1,750km resolution”. They must be meters. This has been corrected in the text. We added the total area of the region as well, 2380km^2^ (line 127).

6. ***Lines 140 – 144 : describe how did you distribute 384 over the study region (what is the relationship between 384 people and 440 square areas?)***

Answer: We improved our explanation in lines 132-152. We used a regular sampling design to distribute uniformly our participants in our study region. We divided the whole study area using a regular grid. One household was selected by defined area, the one closest to the centroid, and then one person between 5 to 15 years of age and one person older than 15 years old were randomly selected and invited to take part in the study. In case of participation declining, the following household closest to the centroid was selected and so forth. The resultant distribution of our participants it is shown in Figure S1. The required number of participants was 440 participants younger than 15 years old and 440 participants older than 15 years old. As explained in comment 4, we estimated a sample size of 384 participants: we assumed a conservative 50% prevalence in both age groups to ensure a sample size enough to achieve a precision of 5% in the estimation of the 95% CIs regardless of the true prevalence. Moreover, we assumed 10% of loss to follow-up or participation rejection in all square area. Due to equal division for regular sampling in the study area this 10% arised approximately 13%. Thus, 440 participants younger than 15 years old and 440 participants older than 15 years old were required.

7. ***Line 222: explain the socioeconomic variable you considered in the study and how SES was developed.***

Answer: We explained with further detail how the socioeconomic variable was built in lines 241-250. The text is the following: “A socioeconomical wealth index was built based on household characteristics and assets to attribute a household socioeconomic score (SES) to each household of Manhiça district. For that, we performed a multiple correspondence analysis (MCA) to determine the weights of every characteristic or asset and 17 household characteristics and assets were included (Ayele et al 2014). We excluded water and sanitation variables to account them separately, since they are key factors of STH infection (Strunz et al 2014). Manhiça district household SES was then divided in tertiles to classify households in poor socioeconomical status, middle socioeconomical status and rich socioeconomical status during participants’ description. SES continuous variable was also kept to be used during statistical modelling of infection.”

8. ***Line 254 : provide a reference for considering correlation coefficient of 0.8 as the cut off***

Answer: We arbitrarily defined a threshold of 0.8 in Pearson's correlation coefficient to ensure the minimum collinearity in our model. We improve the text in lines 279 - 283: “Collinearity was explored using cross-correlations among explanatory variables. To address the issue of collinearity in our model, if a pair of variables had a Pearson's correlation coefficient greater than 0.80, only one of two them was retained in the model.”

9. ***Lines 270 – 272: explain why did you fit a negative binomial model in this study and provide details of the fitted model including considered covariates, assessment of non-linear associations in the covariates, model fit statistics, was there any over dispersion, if so did you consider generalized mixed effect model and assessed the necessity of random effects?***

Answer: We recognise that there was an error in naming our model in the discussion of this manuscript. This has been corrected. Although many models were tested, the final model used in this manuscript is a binomial generalized linear model. We believe that using a negative binomial generalized linear model when modelling quantitative results from diagnostics (e.g. egg counts) could account for quantitative results’ overdispersion. In our case, we modelled a positive or negative result to the diagnostic. We had weak evidence of overdispersion in the data. The estimates of the dispersion parameter in a quasi-binomial model by technique were all close to 1: Telemann from one stool (0.99), Telemann from two stools (1.01), single Kato-Katz from one stool (1.04), duplicate Kato-Katz from one stool (1.01), single Kato-Katz from two stools (1.04), duplicate Kato-Katz from two stools (1.03) and qPCR (1.03). In addition, all variograms of the point predictions built for all diagnostic techniques (Telemann from one stool and from two stools, single Kato-Katz from one stool, duplicate Kato-Katz from two stools and qPCR) were contained within the tolerance limits generated under the assumption of spatial independence. We thus concluded that the data do not show any strong evidence of spatial correlation (S3 Figure). For these reasons, we did not to include random effects in our model and we did not pursue any more sophisticated modelling to account for overdispersion. We described details of the fitted model in lines 278-365, including the covariates incorporated in the model. Moreover, we added a supplementary S6 Table were we included the estimated prevalence per region with the associated standard errors.

10. ***S3 Figure: Is the figure legend correct? Why did you fit a the binomial generalized linear model if there is no spatial correlation?***

Answer: We corrected the figure legend, the word “linear” was missing. So, the figure legend now is: “S3 Figure. Assessment of spatial autocorrelation in the residuals. Telemann from one and two stools, single Kato-Katz from one or two stool samples, duplicate Kato-Katz from one or two stools and qPCR variograms after fitting the binomial generalized linear model of at least one STH infection.” We observed in the variograms that there was no residual spatial autocorrelation. Thus, all explanatory variables included in the model adequately explained spatial correlation. For that reason, we fitted a generalized linear model and not a generalized linear mixed model. Further explanation is provided in comment 9.

11. ***Line 273: explain the spatial units***

Answer: We estimated the prevalence of infection for at least one STH per household. For visualization purposes, we obtained neighbourhood prevalences averaging household estimated prevalences per neighbourhood. Thus, the spatial units we chose to visualize prevalences of infection for at least one STH were the neighbourhoods in Manhiça district. This section of the manuscript in lines 306-308 has been modified to: “We obtained estimated neighbourhood prevalences by averaging household level estimated prevalence per neighbourhood. Thus, we used Manhiça district neighbourhoods to visualize the estimated prevalence for at least one STH infection.”

12. ***Results section : authors have done significance testing and provide P values, but have not mentioned which test/s have been used, please include them under methods***

Answer: We apologise that the test we used was only specified in Table 1 but not in methods. We used Chi-square test. We included the section “Participants’ description” under Data analysis in Methods to describe it.

13. ***Lines 302 – 303 : give a breakdown for < 15 and > 15***

Answer: We gave a breakdown, so the sentence is: “Of those study participants owning a latrine, 7.6% had a hygiene station (soap and water) available at the moment of conducting the questionnaire, 6.6% in 5-15 years old and 8.7% in over 15 years old (p-value = 0.342).”

14. ***Lines 319 – 320 and 321 – 322 : evaluate the differences and provide p values***

Answer: We evaluated the differences and we provided the p-values: “ Manhiça district prevalence for at least one STH by single Kato-Katz in one stool was 13.1%, 11.5% in children between 5 to 15 years old and 14.6% in over 15 years old (p-value = 0.188).” and “A third of participants (29.8%) were detected to be hookworm infected for at least one of the techniques, followed by *T. trichiura* infected (13.8%) and *A. lumbricoides* infected (8.1%, p-value < 0.001).”

15. ***Line 372 : explain what is meant by fair correlation***

Answer: We changed “fair correlation” for “good correlation”.

16. ***Fig 3: Legend should be corrected as Log(Fecal egg count) agreement with qPCR Ct value…***

Answer: The editor is right. We rephrased the legend as: “Fig 3. Agreement between qPCR Ct value with faecal egg count logarithm of the four quantitative microscopic methods (single and duplicate Kato-Katz in one stool sample, and single and duplicate Kato-Katz in two stool samples) for *A. lumbricoides*, *T. trichiura* and hookworm. In each graph, the concordance correlation coefficient (ρ) and p-value are provided.”

17. ***Lines 383 – 384: previously authors mentioned that they used 0.8 as the cut off and here mentioned as 0.65, which is correct? (this can be presented in the methods, not under the results)***

Answer: In Methodology, we arbitrarily decided that if two variables had > 0.80 Pearson's correlation coefficient variable, one of them was not included (lines 279-283). In the submitted manuscript, we described in Results that all explanatory variables included showed correlation coefficients below 0.65, and thus, below 0.80. However, this information has been removed to avoid misunderstanding.

18. ***Line 386 – explain what is meant by all variograms and give details (again can include under the methods)***

Answer: We gave detail of the variograms that we built. The paragraph is: “All variograms of the point predictions built for all diagnostic techniques (Telemann from one stool and from two stools, single Kato-Katz from one stool, duplicate Kato-Katz from two stools and qPCR) were contained within the tolerance limits. Thus, we concluded that the data did not show any strong evidence of spatial correlation.” We moved this information from Results to Methods, lines 301-305.

19. ***Figure 4 – what do the white colour areas represent in the map? I can’t see much grey colour regions even though it is mentioned.***

Answer: Map perimeter includes all Manhiça district land. Manhiça district covers populated areas (coloured areas) and unpopulated areas (white areas). At editor’s preference, we could include that in the map legend. We only had a grey neighbourhood in the north of the district. This corresponds to a Sugar cane employees’ residential neighbourhood where nobody participated in the study.

- ***Prevalence estimates can be associated with standard errors and how can you quantify the certainty in the prevalence estimates in these maps?***

Answer: In supplementary S6 Table, we displayed the estimated prevalence per neighbourhood and the associated standard errors.

- ***Suggest to develop maps to show the probability of exceeding/non-exceeding the given WHO thresholds in order to facilitate decision making as this is one of the main objectives of this paper.***

Answer: World Health Organization thresholds were define based on STH infection detected by Kato-Katz in one single stool sample per child. (World Health Organization (2019). Bench Aids for the diagnosis of intestinal parasites. Geneva, World Health Organization.) In this manuscript, we are using different diagnostic methodologies to map the estimated prevalence for at least one STH. Thus, other thresholds than Kato-Katz in one stool should be reconsidered per diagnostic technique. In example, a specific threshold should be defined for qPCR to consider patent infections and discard residual DNA detection (Papaiakovou et al (2019)).

20. ***Line 431 : explain how can you infer an increasing sensitivity tendency if the data does not support***

Answer: We agree that data did not support an increasing sensitivity tendency statistically. We removed that from the text to avoid misunderstanding.

21. ***Lines 433 – 442 : authors have brough up new points which are not mentioned previously. Suggest to include these section under results first and discuss.***

Answer: These points were mentioned in results in lines 402-408: “Regarding Kato-Katz methodologies agreement, the highest agreement with single Kato-Katz from one stool sample was observed in duplicate Kato-Katz from one stool (ρ > 0.90) for all STH. In all three species, this was followed by single Kato-Katz from two stool samples and then duplicate Kato-Katz from two stool samples. In the case of *A. lumbricoides*, all three methodologies showed a ρ > 0.90. (S5 Figure) In addition, single and duplicate Kato-Katz from two stool samples also displayed good agreement between them (ρ > 0.90) in all three species.” They were then discussed in lines 464-473: “Regarding intensity of infection, single Kato-Katz and duplicate Kato-Katz egg count highly agreed, both using one and two stool samples. In this study, samples were thoroughly homogenized before slides were prepared. Slides were read by four different technicians in a blinded way, following good laboratory practice. Furthermore, ten per cent of samples were reread by a senior lab technician. In the laboratory, stool samples were labelled with a unique sample code which could not simply be traced back to the original participant identification number. This in order to prevent any potential bias by the microscopists during the two-slide and two-sample readings. Hence, our results infer that single-slide Kato-Katz would be sufficient if samples are well homogenized, even at low intensities of infection.” We are open for any other suggestion that might help to clarify our results and discussion.

22. ***Line 460: explain what Farrel et al 2018 did.***

Answer: Farrel et al (2018) simulated the output based on single Kato-Katz slides of 41.7 mg. Infection detection sensitivity for Kato-Katz slides increases with the number of eggs in the sample. Thus, they assumed that sampling error in Kato-Katz slides follows a negative binomial distribution with mean egg count for a given person, and that the aggregation parameter is 0.40, based on an analysis of repeated slides from 2083 Ugandan individuals (Coffeng et al 2015). They assumed perfect specificity of Kato-Katz testing as if it was performed by a full-trained technician. In our manuscript, we rephrase lines 555-559 to clarify that: “To solve that, a diagnostic submodel of Kato-Katz egg counting method could be incorporated in the model to account for Kato-Katz poor sensitivity as Farrel et al. 2018 did or estimate with more sensitive diagnostics.” We are open to include further information if the reviewer or editor suggests that.

23. ***Lines 478 : explain nested sampling in the methods.***

Answer: We clarified what we meant by nested sampling. Thus, the sentences has been modified to: “First, Kato-Katz diagnostic methodologies were compared using nested samples, the first egg count of duplicate Kato-Katz is the same to the single Kato-Katz egg count to which it is compared. Even though microscopy reading bias is unlikely as explained above, some residual bias could have occurred.” This is also explained in Methods in lines 260-265.

24. ***Lines 479 – 483: authors need to need to explain this in the methods in details and then discuss.***

Answer: We think that we answered to this comment above, in comment 9.

- ***Anyway, what is meant by geospatial model here?***

Answer: We fitted a generalized linear model here. The model is described in comment 9 and in manuscript between lines 278 and 305. We estimated the prevalences per household and per region using covariates considering the spatial dimension. All variograms of the point predictions built for all diagnostic techniques (Telemann from one stool and from two stools, single Kato-Katz from one stool, duplicate Kato-Katz from two stools and qPCR) were contained within the tolerance limits generated under the assumption of spatial independence. We thus concluded that the data do not show any strong evidence of spatial correlation (S3 Figure) and the covariates adequately explain it. Thus, the inclusion of spatial random effects in our model was not needed. Further explanation is provided in comment 9.

25. ***Lines 495 onwards: Authors mentioned that the geospatial prediction as an essential instrument for targeting STH infection areas and discusses the diagnostic implication in this prediction. However, this has not been adequately discussed under the discussion. Please expand this section.***

Answer: Discussion of this topic has been extended in lines 484-489.

**Reviewer's Responses to Questions**

***<b>Key Review Criteria Required for Acceptance?</b></br></br>***

***As you describe the new analyses required for acceptance, please consider the following:</br></br>***

***<b>Methods</b></br></br>***

***-Are the objectives of the study clearly articulated with a clear testable hypothesis stated?</br>***

***-Is the study design appropriate to address the stated objectives?</br>***

***-Is the population clearly described and appropriate for the hypothesis being tested?</br>***

***-Is the sample size sufficient to ensure adequate power to address the hypothesis being tested?</br>***

***-Were correct statistical analysis used to support conclusions?</br>***

***-Are there concerns about ethical or regulatory requirements being met?</br></br>***

***Reviewer #1: yes***

***Reviewer #2: -Are the objectives of the study clearly articulated with a clear testable hypothesis stated? yes***

***-Is the study design appropriate to address the stated objectives? yes***

***-Is the population clearly described and appropriate for the hypothesis being tested? need to improve***

***-Is the sample size sufficient to ensure adequate power to address the hypothesis being tested? need to improve***

***-Were correct statistical analysis used to support conclusions? need more details***

***-Are there concerns about ethical or regulatory requirements being met? no***

***Please see comments for details.***

***Reviewer #3: Methods: The field collections performed in this study were extensive and the sampling design appears to be sound. However, more information needs to be included regarding the molecular assays used. The primers and probes used were from previous publications and are referenced in the text. A brief description of the method is still needed: I recommend providing the primer and probe sequences used in a table, as well as the thermocycling protocol used. Also, the method used for quantification needs to be described. This is particularly important as it is compared to FEC later in the manuscript.***

Answer: We appreciate reviewer suggestions. However, each of the PCR targets used in this study has been extensively described and published before, including details about protocols and validation steps. In other words, the two PCR multiplex panels applied is this study, are not new. This list of publications, together with the primer and probe sequences, has already been summarized in a table in the given reference (Kaisar et al., 2017). We think that duplicating this information is superfluous.

***The use of a composite reference standard in this context is also unclear. This method is generally applied to characterize the performance of a new method when there is no "gold standard" reference and in these cases, the composite results of the comparator tests are considered to reflect the "true" disease state. It is generally recognized that this method introduces bias and models that estimate sensitivity and specificity correct for this. The issue here is that the specificity of all three tests are assumed to be 100%. PCR-based tests, while quite sensitive, can also have a high number of false positives, particularly when used for screening in low-prevalence populations.***

Answer: We agree with the reviewer that a composite reference standard assumes each of the included diagnostic test to be 100% specific. However, we are convinced that with the right PCR set-up (such as a fully closed real-time system, separate dedicated PCR rooms, proper technical validation, sufficiently implemented quality assessment, the inclusion of appropriate negative controls and optimal performance in an external quality assessment scheme), such a high specificity can be claimed. We have no indications that false positive outcomes are an issue at our PCR set-up. We hope that the adaptations made in the text (line 195-210) clarify this.

**--------------------**

**<b>Results</b></br></br>**

**-Does the analysis presented match the analysis plan?</br>**

**-Are the results clearly and completely presented?</br>**

**-Are the figures (Tables, Images) of sufficient quality for clarity?</br></br>**

**Reviewer #1: yes**

**Reviewer #2: -Does the analysis presented match the analysis plan? yes**

**-Are the results clearly and completely presented? yes**

**-Are the figures (Tables, Images) of sufficient quality for clarity? yes**

**Please see comments for details.**

**Reviewer #3: What is referred to as a composite reference standard in this study seems to actually be a panel diagnostic/screening test. This is fine but more information regarding the specificity of the tests needs to be included. If the authors wish to truly compare the performance of the qPCR panel against the composite reference standard of Telemann and Kato-Katz, more work will need to done to provide a convincing argument, including figuring out how to account for false positives. Overall, the figures are clearly presented**

Answer: See reply above.

***--------------------***

***<b>Conclusions</b></br></br>***

***-Are the conclusions supported by the data presented?</br>***

***-Are the limitations of analysis clearly described?</br>***

***-Do the authors discuss how these data can be helpful to advance our understanding of the topic under study?</br>***

***-Is public health relevance addressed?</br></br>***

***Reviewer #1: n/a***

***Reviewer #2: -Are the conclusions supported by the data presented? yes***

***-Are the limitations of analysis clearly described? need to improve***

***-Do the authors discuss how these data can be helpful to advance our understanding of the topic under study? yes***

***-Is public health relevance addressed? need to improve***

***Please see comments for details.***

***Reviewer #3: The authors do a good job of describing how the use of highly sensitive tests methods can be incorporated socioeconomic and demographic data can better inform STH disruption strategies. However, the problem of false positive results with qPCR testing needs to be addressed.***

Answer: See reply above.

***--------------------***

***<b>Editorial and Data Presentation Modifications?</b></br><br/>***

***Use this section for editorial suggestions as well as relatively minor modifications of existing data that would enhance clarity. If the only modifications needed are minor and/or editorial, you may wish to recommend “Minor Revision” or “Accept”.***

***Reviewer #1: (No Response)***

***Reviewer #2: Requies major revisons***

Answer: We hope we have addressed these issues.

***Reviewer #3: I think this study is well done and worthy of publication in NTD once these test-related issues are resolved. One additional note: In a few places in the manuscript "low intensity settings" are mentioned when I think what is meant are low prevalence settings. The terms are often interchanged but it gets confusing when talking about high/low infection intensities (in individuals) and infection prevalence (in a population) in the same paper.***

Answer: Areas where continued MDA has been implemented might tend to have low intensities of infection. MDA with anthelmintics might have reduced burden of infection, and thus, morbidity caused by STH infection. However, after frequent MDA, control programs might aim for transmission interruption. To confirm transmission interruption, sensitive diagnostic methods to detect low intensity infections are crucial. This is the reason why we focus in low intensity settings. We hope this explanation have clarified reviewer doubts.

***--------------------***

***<b>Summary and General Comments</b></br></br>***

***Use this section to provide overall comments, discuss strengths/weaknesses of the study, novelty, significance, general execution and scholarship. You may also include additional comments for the author, including concerns about dual publication, research ethics, or publication ethics. If requesting major revision, please articulate the new experiments that are needed.***

***Reviewer #1: While the research question is clear and the methods employed are appropriate, this study lacks of novelty. There is a plethora of studies published in the last 10 years that have addressed this research question using more diagnostic techniques and/or in the context of treatment programs (i.e. using follow up samples). Some of these studies have also been referenced by the authors. Please clarify the novelty and usefulness of this study.***

Answer: World Health Organization aims to eliminate soil-transmitted helminthiases (STH) as a public health problem: reducing to less than 2% the proportion of moderate and heavy infections. Some regions are reaching WHO goals, but transmission could rebound from residual hotspots if interventions are halted without a thorough epidemiological evaluation. For that, sensitive diagnostic methods to detect low intensity infections and localization of ongoing transmission are crucial. In this study, we propose geospatial prediction as an essential instrument for targeting STH infection areas and we evaluate the diagnostic implication in this prediction. To our knowledge, diagnostic implication on distribution of STH infection prediction in low intensity settings in the context of elimination has not been published yet. This manuscript could guide policy make-makers methodology for the confirmation of STH infection transmission interruption.

***Reviewer #2: This study is important as it has evaluated three diagnostic techniques to detect STH infection in a low intensity setting in Southern Mozambique and estimated STH prevalence to identify the locations with ongoing transmission. However, there are a number of issues which require addressing.***

Answer: We hope we have addressed these issues.

***Reviewer #3: TH-associated morbidities place severe health burdens on communities in endemic region. The reduction and elimination of STH infections is a critical need and ongoing epidemiological analyses are needed to assess the efficacy of local mitigation efforts. The authors propose a refinement of diagnostic methods to improve identification of STH infections in low prevalence areas by field sampling households in a rural setting in Southern Mozambique. Participants were enrolled and self-collected stool specimens on two separate days. Stools were analysed using two microscopy-based methods (Telemann, Kato-Katz) and two molecular diagnostic (qPCR) panels. The neighborhood-level prevalence was estimated and the resulting map was overlaid with socioeconomical and environmental data to better understand the spatial distribution of STH infections. Overall, I think that this is an interesting and important study but there are some issues that would need to be corrected prior to acceptance.***

Answer: We hope we have addressed these issues.
