# Supplementary material for: Towards soil-transmitted helminths transmission interruption: The impact of diagnostic tools on infection prediction in a low intensity setting in Southern Mozambique
Source: PLoS Negl Trop Dis. 2021 Oct 25;15(10):e0009803. doi: 10.1371/journal.pntd.0009803 (PMC8568186; doi:10.1371/journal.pntd.0009803)
Supplement: Attachment — Submitted filename: MARS_PLOS_NTDs_Reviewer comments_13082021.docx [file pntd.0009803.s012.docx]

**Editor and reviewer comments**

**Please be sure to consider the concerns about PCR methodology description in your revision. As editor, I agree that a minimum description of the method used improves repeatability and reproducibility and also helps with interpretablity of the outcomes presented here.**

Answer: We appreciate editor comments. We added further details on the multiplex real-time PCR procedures in this study to enhance interpretability of the outcomes presented. Moreover, we added oligonucleotide primers and detection probes for multiplex real-time PCR for the simultaneous detection of soil-transmitted helminths in supporting information, S2 Table.

**Reviewer's Responses to Questions**

**<b>Key Review Criteria Required for Acceptance?</b></br></br>**

**As you describe the new analyses required for acceptance, please consider the following:</br></br>**

**<b>Methods</b></br></br>**

**-Are the objectives of the study clearly articulated with a clear testable hypothesis stated?</br>**

**-Is the study design appropriate to address the stated objectives?</br>**

**-Is the population clearly described and appropriate for the hypothesis being tested?</br>**

**-Is the sample size sufficient to ensure adequate power to address the hypothesis being tested?</br>**

**-Were correct statistical analysis used to support conclusions?</br>**

**-Are there concerns about ethical or regulatory requirements being met?</br></br>**

**Reviewer #2: -Is the study design appropriate to address the stated objectives? yes**

**-Is the population clearly described and appropriate for the hypothesis being tested? yes**

**-Is the sample size sufficient to ensure adequate power to address the hypothesis being tested? yes**

**-Were correct statistical analysis used to support conclusions? need more details**

Answer: Specific answers to reviewer #2 comments on spatial and statistical analysis are provided below.

**-Are there concerns about ethical or regulatory requirements being met? no**

**Reviewer #3: I have to disagree regarding the inclusion of minimum details regarding the qPCR assays used. For the sake of reproducibility and the readers' ability to assess the accuracy of the data, the following questions need to be answered in the methods: For how many cycles were the samples run? What was the Ct cutoff used? Was an internal extraction control used? Primer and probe sequences need to be included in the supplement. It is reassuring that the testing laboratory performs annual proficiency testing, however, this additional information is important as the claim of increased prevalence is entirely based on the performance of these assays.**

**The sentence defining Ct values is unnecessary. The issue is that the assays were not used to quantify parasite specific DNA in this study. If they were, a description of how the Ct was related to quantification would need to be provided (e.g. plotting against a standard curve). The Ct value itself is not a quantitative measure. In this case, the qPCR assays were used qualitatively.**

Answer: We added further details on the qPCR procedure used in this study. In particular, negative and positive control samples were included in each PCR run and no internal extraction control was used. In addition, samples run for 50 cycles and no Ct cut-off was used. Ct value results were analysed using Bio-Rad CFX software (Manager V3.1.1517·0823). Moreover, primer and probe sequences were also included in the supplement (S2 Table). According to reviewer comments, we remove the sentence defining CT values.

**--------------------**

**<b>Results</b></br></br>**

**-Does the analysis presented match the analysis plan?</br>**

**-Are the results clearly and completely presented?</br>**

**-Are the figures (Tables, Images) of sufficient quality for clarity?</br></br>**

**Reviewer #2: Yes**

**Reviewer #3: (No Response)**

**--------------------**

**<b>Conclusions</b></br></br>**

**-Are the conclusions supported by the data presented?</br>**

**-Are the limitations of analysis clearly described?</br>**

**-Do the authors discuss how these data can be helpful to advance our understanding of the topic under study?</br>**

**-Is public health relevance addressed?</br></br>**

**Reviewer #2: -Are the limitations of analysis clearly described? need to improve**

Answer: We specifically answered reviewer #2 comments below.

**-Do the authors discuss how these data can be helpful to advance our understanding of the topic**

**under study? yes**

**-Is public health relevance addressed? need to improve**

Answer: We specifically answer reviewer #2 comments below.

**Reviewer #3: (No Response)**

**--------------------**

**<b>Editorial and Data Presentation Modifications?</b></br><br/>**

**Use this section for editorial suggestions as well as relatively minor modifications of existing data that would enhance clarity. If the only modifications needed are minor and/or editorial, you may wish to recommend “Minor Revision” or “Accept”.**

**Reviewer #2: Minor Revision**

**Reviewer #3: (No Response)**

**--------------------**

**<b>Summary and General Comments</b></br></br>**

**Use this section to provide overall comments, discuss strengths/weaknesses of the study, novelty, significance, general execution and scholarship. You may also include additional comments for the author, including concerns about dual publication, research ethics, or publication ethics. If requesting major revision, please articulate the new experiments that are needed.**

**Reviewer #2: This study is important as it has evaluated three diagnostic techniques to detect STH**

**infection in a low intensity setting in Southern Mozambique and estimated STH prevalence to identify**

**the locations with ongoing transmission. However, there are a still number of issues which require**

**addressing. Please refer my comments in the attached.**

Answer: We specifically answer reviewer #2 comments below.

**Reviewer #3: (No Response)**

**--------------------**

**PLOS authors have the option to publish the peer review history of their article (<a href="https://journals.plos.org/plosntds/s/editorial-and-peer-review-process#loc-peer-review-history" target="_blank">what does this mean?</a>). If published, this will include your full peer review and any attached files.<br><br>**

**If you choose “no”, your identity will remain anonymous but your review may still be made public.<br><br>**

**<b>Do you want your identity to be public for this peer review?</b> For information about this choice, including consent withdrawal, please see our <a href="https://www.plos.org/privacy-policy" target="_blank">Privacy Policy</a>.**

**Reviewer #2: No**

**Reviewer #3: No**

**Authors have answered most of my previous queries and the manuscript has been improved from the last version. However, authors need to clarify and amend below which have not been addressed adequately.**

- **Supplementary figure 1 : please include another map with sampling grid (sampling grid over the map enabling users to get an clear picture of what you did).**

Answer: We edited S1 Figure to complement the explanation of how we design the regular sampling. We included five maps in total. The first three (S1 A, B and C) show the distribution of the households in the district, the grid (1,750m x 1,750m) that divided the district households and the centroid location in each cell of the grid. The other two (S1 D and E) display the distribution of all the households in the district and the location of study participants after conducting regular sampling.

- **Pls move up the Sample size justification ahead of study population and selection (at present this is dissociated and break the flow of paper).**

Answer: We moved up the Sample size justification, ahead of study population and selection as the reviewer suggests.

- **Authors mentioned “ we believed that using a negative binomial generalized linear model when modelling quantitative results from diagnostics (e.g. egg counts) could account for quantitative results’ overdispersion. In our case, we modelled a positive or negative result to the diagnostic. We had weak evidence of overdispersion in the data.**
  - **Then, why did authors used a binomial generalized linear model?**

Answer: The comment mentioned by the reviewer “ we believed that using a negative binomial generalized linear model when modelling quantitative results from diagnostics (e.g. egg counts) could account for quantitative results’ overdispersion” is only a recommendation in a context of disease control programs. In this article, we are comparing the performance of three STH diagnostic techniques when predicting STH prevalence: Telemann, Kato-Katz and qPCR. The units for all three are different. Telemann is a qualitative microscopy technique. Kato-Katz is a quantitative microscopy technique that intensity of infection was determined by eggs per gram. Lastly, qPCR is also a quantitative technique that intensity of infection was determined by Ct values. Thus, we decided to build the same model with the same predictors and only differing on the outcome of the diagnostic technique used. For that, we decided to use the qualitative outcome of the diagnostic technique (positive or negative). We assessed for overdispersion in our qualitative data (positive and negative) and no evidence of overdispersion was found. After all assessment, a binomial generalized linear model was the best model for the qualitative data.

- **Authors concluded that the data do not show any strong evidence of spatial correlation (S3 Figure) and the covariates adequately explain it. Therefore, they did not include spatial random effects in models.**
  - **The question is whether the authors have investigated for non-spatial random effects? If so, explain how you addressed them in the analysis.**

Answer: We did not consider non-spatial random effects.

- **Authors mention “We thus concluded that the data do not show any strong evidence of spatial correlation (S3 Figure). For these reasons, we did not to include random effects in our model and we did not pursue any more sophisticated modelling to account for overdispersion”.**
  - **Does this say there is existence of (non-=spatial) random effects?**

Answer: Our answer in the previous revision of this manuscript should be corrected as “For these reasons, we did not to include spatial random effects in our model and we did not pursue any more sophisticated modelling to account for overdispersion”.

- **Previously I suggested to include maps to show the probability of exceeding/non-exceeding the given WHO thresholds in order to facilitate decision making as this is one of the main objectives of this paper. However, the response from the authors is not clear and answer the query.**
  - **Since authors have develop prevalence maps, suggest to supplement the prevalence maps with probability of exceeding/non-exceeding the given WHO thresholds.**

Answer: We supplemented the prevalence maps with probability of exceeding WHO threshold for mass drug administration (20% of infection). See S8 Figure, included it in “Supporting information”.
