# Supplementary material for: Towards soil-transmitted helminths transmission interruption: The impact of diagnostic tools on infection prediction in a low intensity setting in Southern Mozambique
Source: PLoS Negl Trop Dis. 2021 Oct 25;15(10):e0009803. doi: 10.1371/journal.pntd.0009803 (PMC8568186; doi:10.1371/journal.pntd.0009803)
Supplement: Attachment — Submitted filename: Reviewer comments.docx [file pntd.0009803.s011.docx]

Authors have answered most of my previous queries and the manuscript has been improved from the last version. However, authors need to clarify and amend below which have not been addressed adequately.

- Supplementary figure 1 : please include another map with sampling grid (sampling grid over the map enabling users to get an clear picture of what you did).
- Pls move up the Sample size justification ahead of study population and selection (at present this is dissociated and break the flow of paper).
- Authors mentioned “ we believed that using a negative binomial generalized linear model when modelling quantitative results from diagnostics (e.g. egg counts) could account for quantitative results’ overdispersion. In our case, we modelled a positive or negative result to the diagnostic. We had weak evidence of overdispersion in the data.
  - Then, why did authors used a binomial generalized linear model?
- Authors concluded that the data do not show any strong evidence of spatial correlation (S3 Figure) and the covariates adequately explain it. Therefore, they did not include spatial random effects in models.
  - The question is whether the authors have investigated for non-spatial random effects? If so, explain how you addressed them in the analysis.
- Authors mention “We thus concluded that the data do not show any strong evidence of spatial correlation (S3 Figure). For these reasons, we did not to include random effects in our model and we did not pursue any more sophisticated modelling to account for overdispersion”.
  - Does this say there is existence of (non-=spatial) random effects?
- Previously I suggested to include maps to show the probability of exceeding/non-exceeding the given WHO thresholds in order to facilitate decision making as this is one of the main objectives of this paper. However, the response from the authors is not clear and answer the query.
  - Since authors have develop prevalence maps, suggest to supplement the prevalence maps with probability of exceeding/non-exceeding the given WHO thresholds.
